# A series of four novel alkaline earth metal–organic frameworks constructed of Ca(ii), Sr(ii), Ba(ii) ions and tetrahedral MTB linker: structural diversity, stability study and low/high-pressure gas adsorption properties†‡

**DOI:** 10.1039/d0ra05145d

**Published:** 2020-09-01

**Authors:** Miroslav Almáši, Vladimír Zeleňák, Róbert Gyepes, Ľuboš Zauška, Sandrine Bourrelly

**Affiliations:** Department of Inorganic Chemistry, Faculty of Science, P. J. Šafárik University Moyzesova 11 SK-041 54 Košice Slovak Republic miroslav.almasi@upjs.sk; Department of Inorganic Chemistry, Faculty of Science, Charles University Hlavova 2030 CZ-128 43 Prague Czech Republic; Aix-Marseille University, CNRS, MADIREL Marseille Cedex 20 F-133 97 France

## Abstract

A series of four novel microporous alkaline earth metal–organic frameworks (AE-MOFs) containing methanetetrabenzoate linker (MTB) with composition {[Ca_4_(μ_8_-MTB)_2_]·2DMF·4H_2_O}_*n*_ (UPJS-6), {[Ca_4_(μ_4_-O)(μ_8_-MTB)_3/2_(H_2_O)_4_]·4DMF·4H_2_O}_*n*_ (UPJS-7), {[Sr_3_(μ_7_-MTB)_3/2_]·4DMF·7H_2_O}_*n*_ (UPJS-8) and {[Ba_3_(μ_7_-MTB)_3/2_(H_2_O)_6_]·2DMF·4H_2_O}_*n*_ (UPJS-9) (UPJS = University of Pavol Jozef Safarik) have been successfully prepared and characterized. The framework stability and thermal robustness of prepared materials were investigated using thermogravimetric analysis (TGA) and high-energy powder X-ray diffraction (HE-PXRD). MOFs were tested as adsorbents for different gases at various pressures and temperatures. Nitrogen and argon adsorption showed that the activated samples have moderate BET surface areas: 103 m^2^ g^−1^ (N_2_)/126 m^2^ g^−1^ (Ar) for UPJS-7′′, 320 m^2^ g^−1^ (N_2_)/358 m^2^ g^−1^ (Ar) for UPJS-9′′ and UPJS-8′′ adsorbs only a limited amount of N_2_ and Ar. It should be noted that all prepared compounds adsorb carbon dioxide with storage capacities ranging from 3.9 to 2.4 wt% at 20 °C and 1 atm, and 16.4–13.5 wt% at 30 °C and 20 bar. Methane adsorption isotherms show no adsorption at low pressures and with increasing pressure the storage capacity increases to 4.0–2.9 wt% of CH_4_ at 30 °C and 20 bar. Compounds displayed the highest hydrogen uptake of 3.7–1.8 wt% at −196 °C and 800 Torr among MTB containing MOFs.

## Abstract 2

Four novel microporous alkaline earth metal–organic frameworks (AE-MOFs) containing methanetetrabenzoate linker (MTB): UPJS-6, UPJS-7, UPJS-8 and UPJS-9 have been successfully prepared, characterized and tested as adsorbents for different gases.

## Graphical abstract

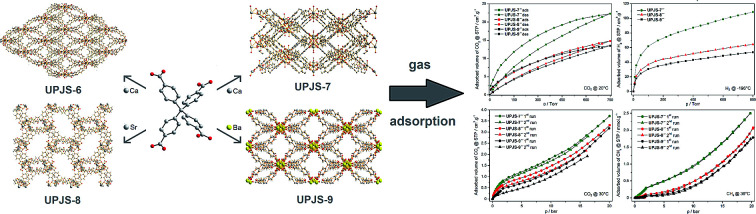

## 1. Introduction

Metal–organic frameworks (MOFs) represent a class of inorganic–organic hybrid materials constructed from metal ions or clusters and organic linkers to form porous polymeric frameworks with interesting properties. Due to their structural and functional diversity, large surface area, porosity and affinity toward various guest molecules, MOFs have been tested as materials for different applications including gas storage and separation,^1^ heterogeneous catalysis,^2^ drug delivery,^3^ magnetic refrigeration^4^ and additives in batteries.^5^ However, gas adsorption was and still is one of the most important applications of MOFs. Hydrogen storage in MOF is currently being studied intensively. Hydrogen as a technologically important gas provides an ideal clean energy source, since no detrimental molecules are produced causing an environmental burden. Hydrogen is the smallest and simplest gas molecule with a very low boiling point, and its storage and transportation present a challenge.^6^ The second technologically important gas is carbon dioxide, which is industrially used to prepare many intermediate or fine chemicals. CO_2_ is also the main component of greenhouse gases and represents one of the largest anthropogenic impacts on the environment. For this reason, it is necessary to reduce its concentration in the atmosphere and thus limit global warming.^7^ Physical absorption of hydrogen and carbon dioxide in porous MOFs represents an interesting approach to the storage of the mentioned gases.

Alkaline earth metal–organic frameworks (AE-MOFs) are investigated relatively less compared to MOFs that contain transition metals, although they display several unique advantages such as: (a) high abundance in earth's crust, (b) low cost, (c) low toxicity (particularly for calcium), (d) low density (Be, Mg, Ca, Sr) that offers gravimetric benefit for storage applications, (e) stability in air and (f) ionic nature leading to strong interactions with negative functional groups such as carboxylate. Unlike the transition metals, which tend to form largely predictable secondary building units (SBUs), the bonds between alkaline earth metal centres with functional groups are predominantly ionic in nature and the prediction and control over coordination geometry is ambiguous. The nature of organic linker plays an important role in the overall dimensionality and topology of the frameworks. Carboxylate-based linkers are those used most commonly to synthesize AE-MOFs due to the strong interaction between carboxylate oxygen atoms and alkaline earth metals. Based on the HSAB theory, AE metals fall under the hard acids and coordinate well with carboxylate functionalities.^8^ Carboxylate linkers with tetrahedral symmetry are highly suitable for MOF preparation, because they form the desired porous frameworks with large surface areas. To date, many MOFs have been prepared that contain ligands that possess tetrahedral geometry: methanetetrabenzoate (MTB),^9^ methanetetrabiphenylcarboxylate,^10^ methanetetrakis(benzene-4,1-diyl)tetraacrylate,^11^ methane tetrakis(benzene-4,1-diyl)tetrakis(ethyne-2,1-diyl)tetrabenzoate^11^ and tetrakis{3,5-bis[(4-carboxylate)phenyl]phenyl}methane.^12^ The chemical and physical properties of AE-MOFs have found applications in gas storage and separation,^13^ catalysis,^14^ sensing^15^ and proton conduction.^16^ Numerous AE-MOFs have been explored predominantly for gas storage of carbon dioxide, hydrogen and also for nitric oxide, noble gases and hydrocarbons.^13^

In our previous studies, we reported several compounds containing tetrahedral methanetetrabenzoate linker (MTB) with Zn(ii),^17,18^ Ni(ii)^19^ and Pb(ii)^20,21^ metal centres and tested them as adsorbents for gases and heterogeneous catalysts. In the course of our continuing investigation into the crystal engineering of MOFs containing MTB linker and their applications, we combined the advantages of AE metals with MTB. Four novel MOFs were successfully prepared, characterized and tested as adsorbents for nitrogen, argon, carbon dioxide, methane and hydrogen at different temperatures and pressures.

## 2. Experimental

### 2.1. Materials

**Fig. 1:**
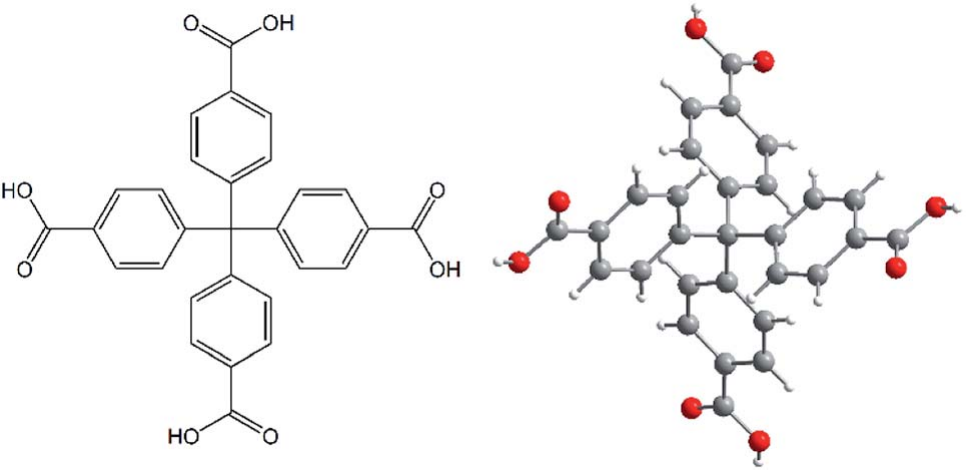
A molecular structure of methanetetrabenzoic acid (H_4_MTB) as an organic building block used in the synthesis of the final MOF compounds.

All chemicals used in the synthesis of AE-MOFs and H_4_MTB (see Fig. 1)^17^ were obtained from Sigma-Aldrich Company or Acros Organics and used without purification.

#### 2.1.1 Synthesis of compounds

The compounds were prepared by solvothermal synthesis in glass Ace® autoclaves. In the synthesis, the nitrates of the respective cations and methanetetrabenzoic acid were used in a molar ratio 5 : 1.20 mg (0.04 mmol) of methanetetrabenzoic acid and 48 mg (0.20 mmol; Ca(NO_3_)_2_·4H_2_O) for synthesis of compounds UPJS-6 and UPJS-7, 43 mg (0.20 mmol; Sr(NO_3_)_2_) for compound UPJS-8 and 53 mg (0.20 mmol, Ba(NO_3_)_2_) for synthesis of compound UPJS-9 were used. H_4_MTB was dissolved in 3 cm^3^ of *N*,*N*′-dimethylformamide (DMF), followed by the addition of the appropriate metal nitrates and then 3 cm^3^ of ethanol and 2.8 cm^3^ of water were added while stirring the solutions until the reactants were completely dissolved. Prepared solutions were sealed in glass autoclaves and the temperature programme was controlled by a thermoregulator. The temperature mode was set as follows: the reaction mixtures of compounds UPJS-6, UPJS-8 and UPJS-9 were heated to 80 °C and UPJS-7 to 110 °C with a heating rate of 0.5 °C min^−1^ for 72 hours. After this time, the reaction vessels were cooled to room temperature with a cooling rate of 0.5 °C min^−1^. Prepared materials were filtered off, several times washed with mixture of solvents used in the synthesis and dried in the stream of air, yields: 17.8 mg of UPJS-6 (yield 38%), 17.5 mg of UPJS-7 (yield 28%), 18.5 mg of UPJS-8 (yield 35%) and 22.5 mg of UPJS-9 (yield 35%). Elemental analysis for UPJS-6 (C_64_H_54_N_2_O_22_Ca_4_; 1363.43 g mol^−1^) clcd: C, 56.38%; H, 3.99%; N, 2.05%; found: C, 57.14%; H, 4.05%; N 1.98%; for UPJS-7 (C_55.5_H_68_N_4_O_25_Ca_4_; 1351.46 g mol^−1^) clcd: C, 49.32%; H, 5.07%; N, 4.15%; found: C, 48.43%; H, 5.10%; N 4.23%; for UPJS-8 (C_55.5_H_66_N_4_O_23_Sr_3_; 1419.99 g mol^−1^) clcd: C, 46.94%; H, 4.68%; N 3.95%; found: C, 46.51%; H, 4.72%; N, 3.96% and for UPJS-9 (C_49.5_H_58_N_2_O_24_Ba_3_; 1476.98 g mol^−1^) clcd: C, 40.25%; H, 3.96%; N 1.90%; found: C, 40.86%; H, 3.87%; N, 2.01%. Assignment of characteristic absorption bands (in cm^−1^) in IR spectra for UPJS-6: *ν*(OH) 3409; *ν*(CH)_ar._ 3069, 3038; *ν*(CH)_aliph._ 2963, 2932, 2891; *ν*(C=O) 1664; *ν*(C=C) 1600; *ν*_as_(COO^−^) 1543; *ν*_s_(COO^−^) 1407; UPJS-7: *ν*(OH) 3423; *ν*(CH)_ar._ 3068, 3040; *ν*(CH)_aliph._ 2996, 2933, 2890; *ν*(C=O) 1670; *ν*(C=C) 1660; *ν*_as_(COO^−^) 1547; *ν*_s_(COO^−^) 1408; UPJS-8: *ν*(OH) 3412; *ν*(CH)_ar._ 3067, 3039; *ν*(CH)_aliph._ 2959, 2931, 2887; *ν*(C=O) 1662; *ν*(C=C) 1598; *ν*_as_(COO^−^) 1541; *ν*_s_(COO^−^) 1398; UPJS-9: *ν*(OH) 3407; *ν*(CH)_ar._ 3064, 3037; *ν*(CH)_aliph._ 2957, 2929, 2884; *ν*(C=O) 1660; *ν*(C=C) 1590; *ν*_as_(COO^−^) 1537; *ν*_s_(COO^−^) 1392 cm^−1^. Corresponding IR spectra of prepared compounds are depicted in Fig. S1 in ESI.‡

#### 2.1.2 Solvent exchange process

To remove DMF molecules from the channel system of prepared materials (UPJS-7, UPJS-8 and UPJS-9) for easier activation of compounds, the solvent exchange process was performed. 100 mg of as-synthesized samples were gently grinding and then dispersed in 30 cm^3^ of ethanol for 5 days, while ethanol was changed every day. Solvent exchanged samples were denoted in the manuscript as UPJS-7′, UPJS-8′ and UPJS-9′.

### 2.2 Characterization of prepared materials

The elemental analysis was performed with a CHNOS Elemental Analyzer vario MICRO from Elementar Analysensysteme GmbH with ±2–3 mg of sample.

The infrared spectra were recorded with Avatar FT-IR 6700 spectrometer in the range 4000–400 cm^−1^. The samples were prepared in the form of a KBr pellet with a complex/KBr mass ratio of 1/100. Before IR measurements KBr was dried at 700 °C for 3 h in an oven and cooled in a desiccator.

Thermal behaviour of as-synthesized and solvent exchanged samples was studied by thermogravimetry analysis (TGA) in the temperature range of 30–800 °C. The sample with a weight of approximately 20 mg was placed in Al_2_O_3_ crucible and heated under a dynamic atmosphere of air (60 cm^3^ min^−1^) with a heating rate of 10 °C min^−1^, using the TGA Q500 apparatus.

HE-PXRD measurements were carried out at the P01 wiggler beamline of the PETRA III positron storage ring in DESY (Hamburg, Germany). The patterns were collected during *in situ* heating with a heating rate of 10 °C min^−1^ in an atmosphere of argon and beam energy of 60 keV, corresponding to a wavelength of 0.207150 Å. The diffracted photons were collected using an image plate detector PerkinElmer (2300 × 2300 pixels, each pixel having a size of 150 × 150 μm^2^) carefully aligned orthogonal to the X-ray beam. The sample-to-detector distance and the detector tilt were determined from diffraction patterns obtained from CeO_2_ standard reference material. The two-dimensional diffraction patterns were integrated using the QXRD software. The observed X-ray scattering intensities were corrected for background, absorption, polarization, incoherent and multiple scattering effects.

### 2.3 Single-crystal X-ray diffraction (SC-XRD) experiments

Single-crystal diffraction data for UPJS-6 (CCDC: 1978638‡), UPJS-8 (CCDC: 1978639‡), UPJS-9 (CCDC: 1978637‡) were collected on a Nonius Kappa four-circle CCD diffractometer equipped with an Apex II (Bruker) detector at 150 K and for UPJS-7 (CCDC: 1978636‡) on a Bruker D8 VENTURE diffractometer at −153 °C. Data reduction in all cases was carried out by diffractometer software. The phase problem was solved by intrinsic phasing and structure models were refined by full-matrix least-squares on F^2^ using the Shelx program suite.^22^ Nonhydrogen atoms were refined anisotropically. Hydrogen atoms were included in idealized positions and refined isotropically. The contribution of guest molecules located in the pores was subtracted by the SQUEEZE procedure in Platon.^22^ The figures of final crystal structures were drawn using the DIAMOND software. Detailed crystallographic data are given in Table S1 in ESI‡ and selected bond lengths (Å) and angles (°) for all compounds are summarized in Tables S2–S5 in ESI.‡

### 2.4 DFT computations

DFT computations have been carried out using Gaussian 16, Revision A.03. Computations employed the M11 functional and the 6–31 + G(d,p) basis set for all atoms.^23^ The Hessians for geometry optimizations were estimated before the initial step of optimizations.

### 2.5 Gas adsorption measurements

Nitrogen adsorption isotherms measured at −196 °C were taken using Quantachrome Nova 1200e automatic adsorption analyser. Before the adsorption studies, the solvents located in the channel system of samples were exchanged by ethanol (for procedure see Section 2.1.2 Solvent exchange process) and further, the samples were activated by the evacuation at 100 °C for 24 h to remove the guest molecules. Activated samples were denoted as UPJS-7′′, UPJS-8′′ and UPJS-9′′. The nitrogen adsorption isotherms for desolvated samples were collected in a relative pressure range from *p*/*p*_0_ = 0.005 to 0.95. Based on the nitrogen adsorption isotherm, the BET specific surface area (*S*_BET_) and pore volume (*V*_p_) of the samples were calculated.

Argon adsorption isotherms were measured at −186 °C, hydrogen adsorption isotherms at −196 °C and carbon dioxide adsorption isotherms were performed at 20 °C using ASAP 2020 apparatus.

The gas adsorption measurements at 30 °C and pressures up to 20 bar were performed with CO_2_ and CH_4_ using a homemade high-throughput instrument.^24^ Gas adsorption experiments were measured *via* a manometric gas dosing system on three samples in parallel. The amounts of gas adsorbed were calculated by an equation of state using the Reference Fluid Thermodynamic and Transport Properties (REFPROP) software package 8.0 of the National Institute of Standards and Technology (NIST).^25^ The high pressure adsorption isotherms were measured on the same batch of the sample as nitrogen adsorption. Samples were thermally activated individually *in situ* under the primary vacuum, at 80 °C overnight.

The regenerability of the samples has been evaluated under mild conditions. For each gas (CO_2_ and CH_4_) two measurements with the same parameters were performed on the same sample. Between the first and the second experiment, the samples were submitted to an evacuation step at 30 °C and under the primary vacuum during 80 minutes. In this way, from the second gas adsorption measurement, the regenerability/recovery of the samples was checked under the mentioned conditions.

## 3. Results and discussion

### 3.1 Synthesis

All the compounds investigated in the present study were prepared using a solvothermal synthesis under mild synthetic conditions (80–110 °C) and in a molar ratio of 5 : 1. It is known that the synthesis of MOF may be included in so-called combinatorial synthetic chemistry since the successful preparation of porous coordination polymers depends on many factors such as: reactant concentration, the presence of a co-solvent, solution pH, metal to ligand ratio, properties of the used counterion, reaction temperature, and reaction time, can have a considerable impact on the final composition of the products.^26^ In the case of the prepared calcium(ii) compounds UPJS-6 and UPJS-7, the influence of the reaction temperature was observed. In the synthesis of these materials, the same reaction conditions were used except temperature. Compound UPJS-6 was prepared at 80 °C and represents the low-temperature phase and compound UPJS-7 was synthesized at 110 °C, which can be referred as the high temperature form. The temperature difference of 30 °C had a significant effect on the mutual arrangement of the building components, which led to the formation of different polymeric frameworks with different porosity (see description of crystal structures below).

The exact formula and composition of all prepared samples were established based on the combination of single-crystal X-ray diffraction experiments, elemental analysis and thermogravimetric analysis results.

### 3.2 Crystal structures

#### 3.2.1 Crystal structure of {[Ca_4_(μ_8_-MTB)_2_]·2DMF·4H_2_O}_*n*_ (UPJS-6)

**Fig. 2:**
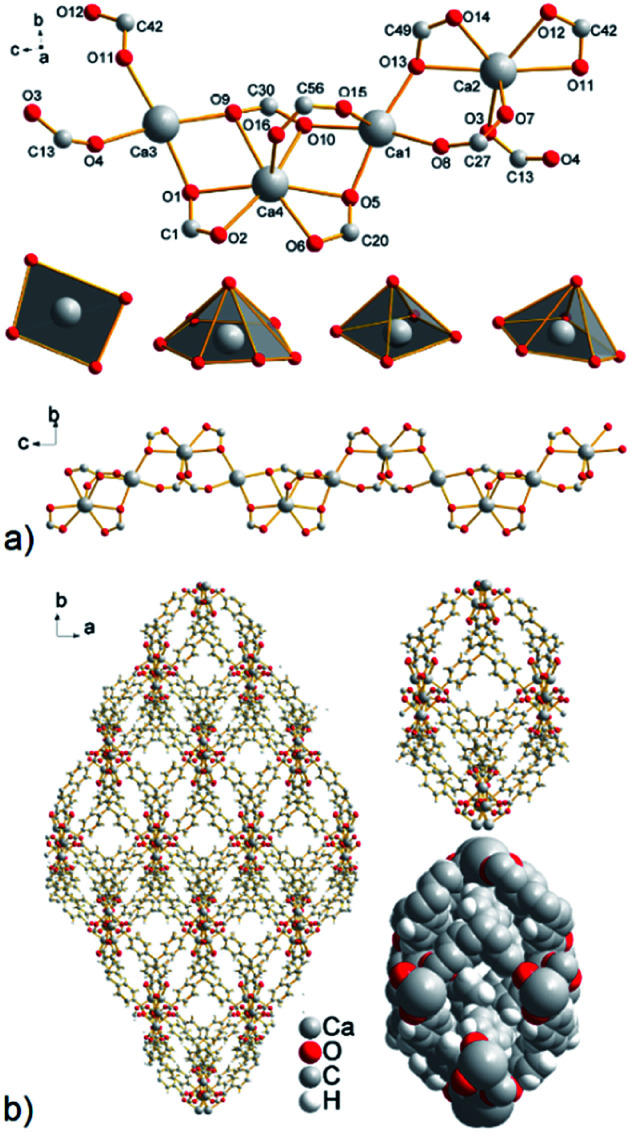
(a) A view of four crystallographically independent calcium ions with the corresponding shape of coordination polyhedra and final 1D sinusoidal calcium chain propagating along the *c* crystallographic axis. (b) The resulting 3D motif in UPJS-6 with closed/isolated cages.

In the crystal structure of compound UPJS-6, four crystallographically independent calcium cations with different coordination numbers and coordination polyhedra are present (see Fig. 2a). The central atom Ca3 with [CaO_4_] donor set is coordinated by four oxygen atoms O1, O4, O9 and O11 from the four different coordinated carboxylate groups of MTB^4−^ ligands. The oxygen atoms O1 and O9 originated from two COO^−^ groups with the carbonyl atoms C1 and C30 which are coordinated in the chelate-*anti*–*anti* mode and two oxygen atoms O1 and O3 from carboxylates C13 and C42 are coordinated in *syn*–*syn* mode. The resulting polyhedron geometry of Ca3 could be described as a deformed planar square (see Fig. 2a), since the angles around the calcium atom significantly differ from the ideal 90° and are in the range of 95.71(15)–76.08(13)°. The second central atom, Ca4 with [CaO_7_] donor set is heptacoordinated by oxygen atoms of four carboxylate moieties coordinated in *syn*–*syn* (carbonyl atom C56), chelate-*anti* (C1, C20) and chelate-*anti*–*anti* (C30) fashion and the coordination polyhedron around Ca4 could be described as a deformed hexagonal pyramid. Since the hexagonal basal plane is made up of three chelate-coordinated carboxylate ligands whose bite angle is limited, the angles of O–Ca–O differ from an ideal value of 60° for a hexagon. As an example, the chelate angle O5–Ca4–O6 having the highest value of 52.65(12)° (see Table S2 in ESI‡). Described carboxylate groups make a bridge between Ca3, Ca4 and Ca1 central atoms. The coordination polyhedron of Ca1 atom could be described as a distorted square pyramid with [CaO_5_] donor set. The basal plane of the polyhedron consists of O8, O5, O10 and O13 oxygen atoms from three carboxylate groups coordinated in chelate-*anti* mode (carbonyl atoms C20, C30 and C49) and one carboxylate group in *syn*–*syn* (C27) mode. Oxygen O15 of the second *syn*–*syn* (C56) carboxylate group completes the pyramidal top of the polyhedron. Carboxylate groups with carbonyl atoms C49 and C27 form a bridge between the calcium ions Ca1 and Ca2. The central atom Ca2, in contrast to the other crystallographically independent calcium ions, is six coordinated with deformed pentagonal pyramidal geometry (see Fig. 2a) and [CaO_6_] donor set. The basal plane of the polyhedron consists of oxygen atoms O3, O11, O12, O13, O14 and oxygen atom O7 decorates the top of the pentagonal pyramid. The central atoms in the polymeric cluster [Ca_4_(μ_2_-COO)_7_(μ_3_-COO)] are arranged in the Ca3–Ca4–Ca1–Ca2 sequence with Ca⋯Ca distances: 3.8746(16) Å (Ca3⋯Ca4), 3.7481(16) Å (Ca4⋯Ca1) and 4.1509(17) Å (Ca1⋯Ca2) (see Table S2 in ESI‡). The described geometric arrangements of the coordinated carboxylate groups on the central atoms propagated along the *c* crystallographic axis to form the sinusoid-like 1D polymer chains as shown in Fig. 2a (bottom).

The SC-XRD analysis showed that in the crystal structure of compound UPJS-6 isolated cages are present. Although the inner diameter of the cage is relatively large with a size of 18.3 × 8.4 Å^2^, the inlet openings are only 0.7 × 0.5 Å^2^ in size (see Fig. 2b). The total free volume in the structure of compound UPJS-6 analysed by the PLATON program is 3434.1 Å^3^ and represents 44.1%, with respect to the total volume of the unit cell (7789.5 Å^3^). Analysis of the framework using the ToposPro program showed that the compound did not imitate any known mineral and the skeleton topology could be designated as 3,4^2,6,8-c net.^27^ Since compound UPJS-6 contains isolated pores in the crystal structure, its gas adsorption properties were not studied.

#### 3.2.2 Crystal structure of {[Ca_4_(μ_4_-O)(μ_8_-MTB)_3/2_(H_2_O)_4_]·4DMF· 4H_2_O}_*n*_ (UPJS-7)

**Fig. 3:**
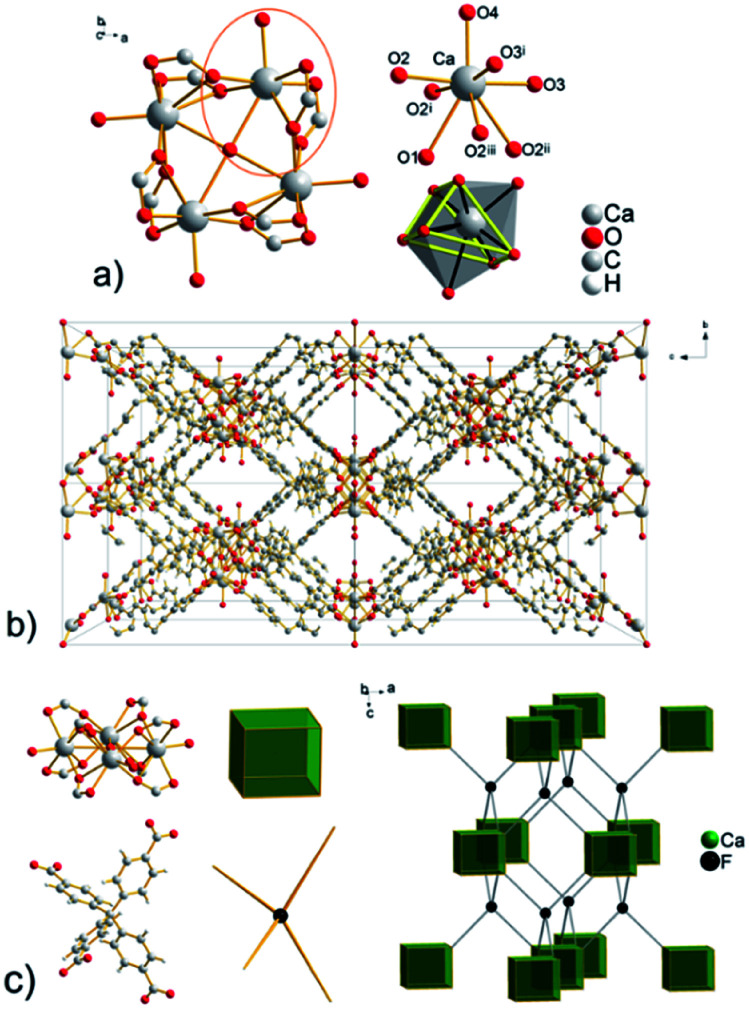
(a) Cyclotranuclear Ca(ii) cluster with composition [Ca_4_(μ_4_-O)(μ_2_-COO)_8_(H_2_O)_4_], the coordination environment of Ca(ii) ions and a view of bicapped trigonal prismatic polyhedron ((i) *x*, *y*, 2 − *z*; (ii) −*x*, *y*, 2 − *z*; (iii) −*x*, *y*, *z*). (b) View of the framework in UPJS-7 along *a* = *b* crystallographic axis. (c) Topological analysis of the framework with a demonstration of the CaF_2_ structure imitation.

Compound UPJS-7 (see Fig. 3) consists of an interesting square planar tetranuclear cluster with composition [Ca_4_(μ_4_-O)(μ_2_-COO)_8_(H_2_O)_4_], in which four Ca ions share a common O1 oxygen atom (μ_4_-binding), which is located in a special position on a four-fold axis. It is of interest for this cluster that the bridge oxygen atom is not tetrahedrally surrounded, as in various modifications of the MOF-5 compounds (IRMOFs^28^), basic beryl or zinc acetate. The axially coordinated oxygen atoms are located in the corners of the square planar cluster with O–Ca–O angle of 90° and the distance between central atoms Ca⋯Ca is 3.797(4) Å (see Table S3 in ESI‡). Eight carboxylate groups in [Ca_4_(μ_4_-O)(μ_2_-COO)_8_(H_2_O)_4_] cluster are coordinated in chelate-*anti* coordination mode and the secondary building unit have a cubic shape (see Fig. 3c). Calcium cations are eight-coordinated with donor set [CaO_8_] and the shape of the coordination polyhedron can be described as a bicapped trigonal prism (see Fig. 3a). The bond lengths of coordinated carboxylate groups to Ca ions are 2.320(4) and 2.387(4) Å. The oxygen atom O2 makes a bridge between two calcium ions and the Ca–O bond length is longer, 2.626(4) Å (see Table S3 in ESI‡). The Ca–O2 and Ca–O3 bond lengths of chelating carboxylate groups are 2.320(4) Å and 2.387(4) Å. The bond length of O2 atom with the second calcium atom (Ca^ii^ (ii) −*x*, *y*, 2 − *z*, *anti*-coordination) is longer, 2.626(4) Å (see Table S3 in ESI‡) and is comparable with μ_4_-O coordinated oxygen coordinated to Ca atom (2.685(3) Å (Ca–O1)). The coordination sphere of each central atom is completed by a coordinated water molecule with a Ca–O4 bond length of 2.274(9) Å. The oxygen atoms of the coordinated aqua and oxido ligands form the pyramidal caps of the trigonal prism. In the framework of UPJS-7 with two types of openings are present having a size of 12.6 × 9.4 Å^2^ and 9.4 × 8.0 Å^2^ propagating along *a* and *b* crystallographic axis (see Fig. 3b).

The topological analysis (see Fig. 3b) of the polymeric framework using the ToposPro program^27^ showed that imitates the fluorite (flu, CaF_2_) structure. The central carbon atom of the MTB ligand can be considered the F vertex and the [Ca_4_(μ_4_-O)(μ_2_-COO)_8_(H_2_O)_4_] cluster decorates the calcium(ii) positions in the CaF_2_ structure.

The total free space in the crystal structure of UPJS-7, after removal of the guest molecules and analysed by PLATON, is 2529 Å^3^ (cell volume 4689.0 Å^3^) and corresponds to 57.7%.

#### 3.2.3 Crystal structure of {[Sr_3_(μ_7_-MTB)_3/2_]·4DMF·7H_2_O}_*n*_ (UPJS-8)

**Fig. 4:**
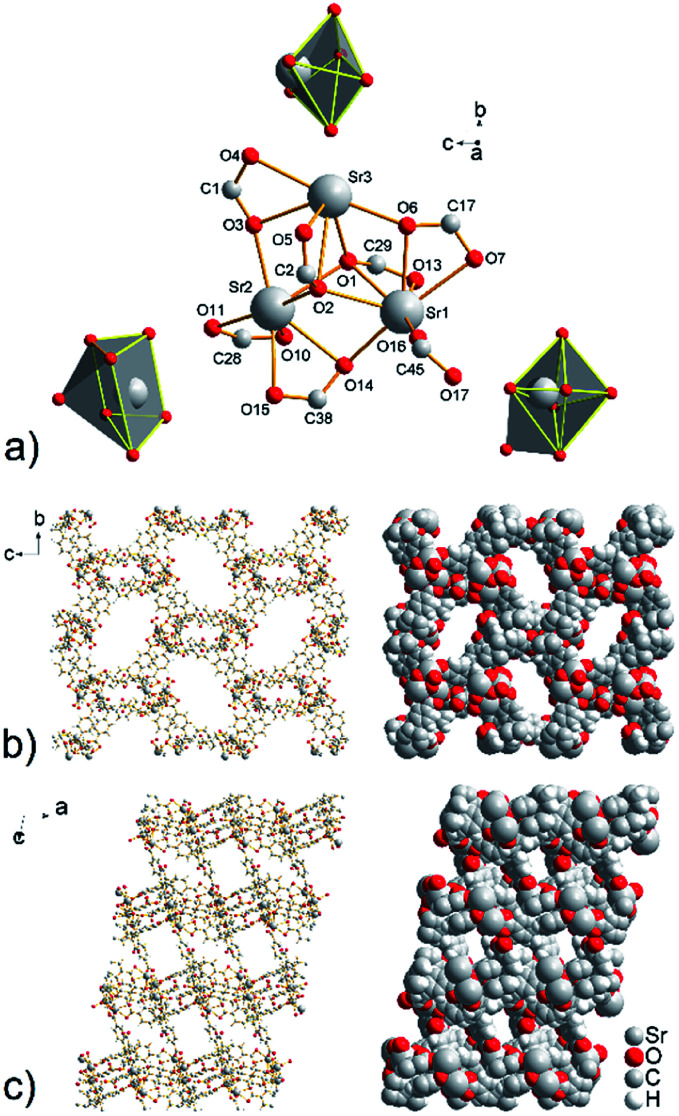
(a) View of the cyclic triangular strontium cluster with composition [Sr_3_ (μ_3_-COO)_2_(μ_2_-COO)_2_(COO)_2_] and the corresponding coordination polyhedra of crystallographically independent Sr(ii) ions. A final crystal packing of the framework with 2D cavities propagating along (b) *a* and (c) *b* axis.

In the crystal structure of compound UPJS-8, three crystallographically independent strontium(ii) ions are present which form the isolated triangular cluster with composition [Sr_3_(μ_3_-COO)_2_(μ_2_-COO)_2_(COO)_2_] (see Fig. 4a). The distances between strontium ions are 3.942(6) Å for Sr1–Sr2, 3.907(5) Å for Sr2–Sr3 and 3.958(6) Å for Sr3–Sr1. The secondary building unit (SBU) has a seven-fold connectivity and the SBU's shape is deformed pentagonal bipyramid. The interesting feature of described coordination polymer is in coordination mode of the carboxylate ligands labeled as C2 and C29, which oxygen atoms O1 and O2 bridged by three different Sr(ii) ions. Thus, oxygen atoms O1 and O2 provide all possible electron pairs to form coordination bonds. The bond distances of μ_3_-coordinated oxygen atoms and strontium ions are in the range of 2.573(3) Å to 2.802(3) Å (see Table S4 in ESI‡). Searching the CCDC database, it can be concluded that the observed coordination mode of the carboxylate group is rare and was commonly found in silver coordination compounds.^29^ The same coordination fashion has so far been observed only in four strontium coordination compounds containing benzene-1,2-dicarboxylate, 2-oxidobenzoate and bis(sulfonyl-4,4′-dibenzoate) ligands.^30^

Strontium cations are in the structure of compound UPJS-8 hepta- and hexacoordinated. The central atom Sr1 with [SrO_7_] donor set is coordinated by the seven oxygen atoms derived from the four different carboxylate moieties of the MTB^4−^ ligand and the shape of the coordination polyhedron can be described as a deformed monocapped octahedron. Strontium ion Sr2 is also heptacoordinated by the seven oxygen atoms, but the shape of the coordination polyhedron [SrO_7_] is a deformed monocaped trigonal prism. The last strontium ion Sr3 is hexacoordinated with [SrO_6_] donor set and the shape of the coordination polyhedron is deformed octahedron (see Fig. 4a). The Sr–O bond distances ranging from 2.485(2) Å (Sr3–O6) to 2.802(3) Å (Sr3–O2) (see Table S4 in ESI‡). The bond lengths of the tridentate-bridged oxygen atoms Sr2–O2 (2.707(3) Å) and Sr3–O2 (2.802(3) Å) are slightly longer compared to the other Sr–O bonds (see Table S4 in ESI‡), due to the electron-withdrawing from the oxygen atoms as a result of the formation of three coordination bonds. The elongated bond distances are consistent with the bond lengths for other strontium coordination polymers containing μ_3_-coordinated carboxylate ligand.^30^

By described coordination of strontium ions and MTB molecules, 3D coordination polymer with the composition of {[Sr_3_(μ_7_-MTB)_3/2_]}_*n*_ is formed. The polymeric framework contains 2D cavities with sizes of 15.5 × 5.0 Å^2^ propagating along *a* crystallographic axis and smaller openings with dimensions of 5.8 and 3.5 Å^2^ propagating along *b* axis (see Fig. 4b and c). The total free volume in the crystal structure of compound UPJS-8 is 55.8% (5242.4 Å^3^ free volume of 9398.2 Å^3^ unit cell volume). Using the ToposPro program,^27^ an analysis of the polymeric framework was performed, which showed that the compound does not imitate any known mineral and is unique. The analysis showed that the framework has a 4,5^2,6,8-c net topology.

#### 3.2.4 Crystal structure of {[Ba_3_(μ_7_-MTB)_3/2_(H_2_O)_6_]·2DMF·4H_2_O}_*n*_ (UPJS-9)

**Fig. 5:**
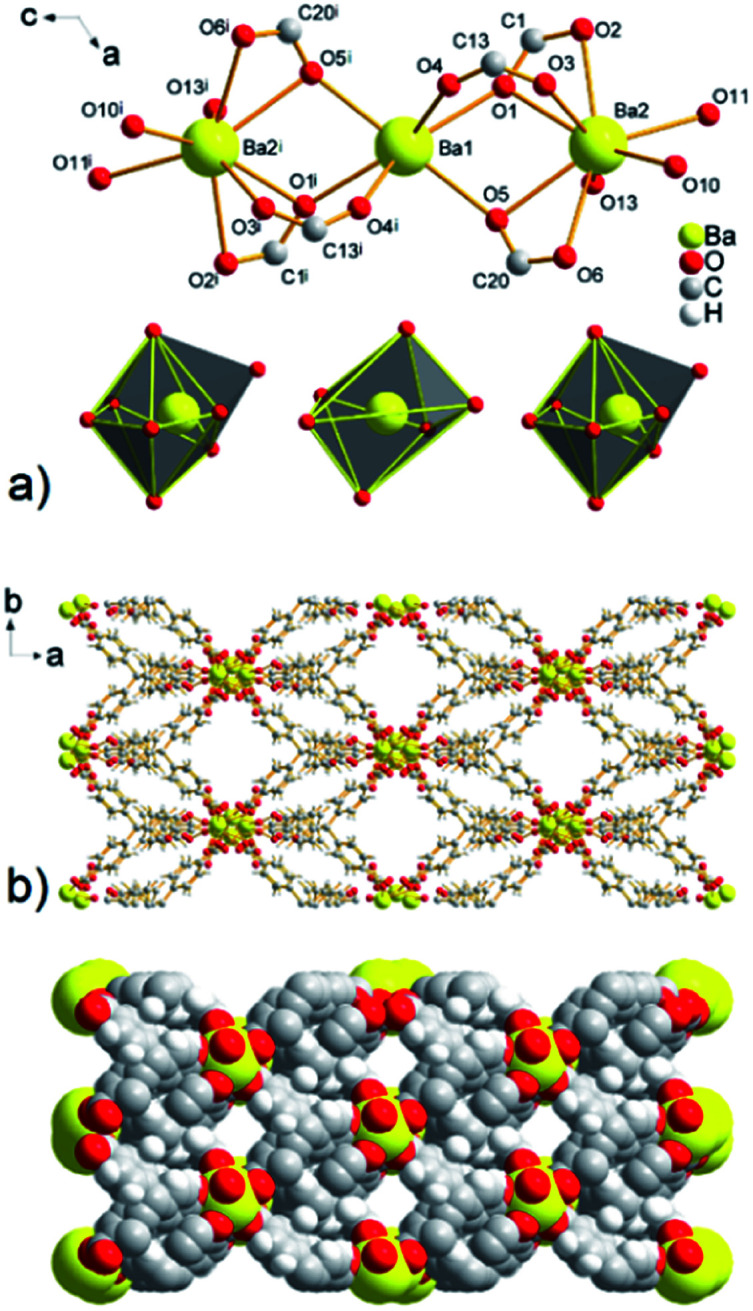
(a) A view of the linear three-nuclear barium cluster with composition [Ba_3_(μ_2_-COO)_6_(H_2_O)_6_] in UPJS-9 (hydrogen atoms were omitted for clarity, (i) = −*x*, *y*, 1/2 − *z*). (b) View of a 3D polymeric framework with 1D channels propagating along *c* crystallographic axis.

A three-nuclear cluster is present in the structure of UPJS-9, similar to compound UPJS-8, but three barium(ii) ions form a linear cluster with the composition [Ba_3_(μ_2_-COO)_6_(H_2_O)_6_]. The cluster is composed of two crystallographically independent barium ions Ba1 and Ba2 which are in the structure of compound UPJS-9 arranged in the Ba2–Ba1–Ba2^i^ sequence (where (i) −*x*, *y*, 1/2 − *z*). Ba2–Ba1 are bridged together by three carboxylate ligands: two coordinated in chelate-*anti* mode (C1, C20) and one carboxylate group in *syn*–*syn* mode (C13). The central atoms are located in a special position and lying on a *c* slide plane (see Fig. 5a). The distance between the two crystallographically independent Ba(ii) ions in the cluster [Ba_3_(μ_2_-COO)_6_(H_2_O)_6_] is 4.4208(4) Å. The described coordination of the carboxylate ligands on the central atoms forms a secondary building unit (SBU) with a connectivity of six and deformed tetragonal bipyramid shape. The central Ba1 atom is hexacoordinated by oxygen atoms with [BaO_6_] donor set and the shape of a polyhedron could be described as deformed tetragonal bipyramid (see Fig. 5a). The coordination sphere of Ba1 consists of four oxygen atoms (O5, O5^i^, O1, O1^i^, where (i) = −*x*, *y*, 1/2 − *z*) originated from coordinated carboxylate groups in chelate-*anti* mode and two oxygen atoms O2 and O2^i^ from two carboxylate groups coordinated in *syn*–*syn* fashion. The carboxylate functional groups (C1, C13, and C20) also form a bridge between Ba1 and Ba2. The coordination sphere of the central atom Ba2 is formed of eight oxygen atoms: three oxygen atoms of coordinated terminal aqua ligands (O10, O11, O13) with bond length range from 2.807(3) to 2.849(5) Å, oxygen atoms O1, O2, O5, and O6 derived from the carboxylate groups coordinated in the chelate-*anti* mode and oxygen O3 from carboxylate coordinated in *syn*–*syn* fashion. The bond lengths of the carboxylate oxygen atoms and Ba2 are comparable to those of coordinated aqua ligands (see Table S5 in ESI‡). The coordination polyhedron of Ba2 ion could be described as a deformed monocapped pentagonal bipyramid with [BaO_8_] donor set, as shown in Fig. 5a.

The combination of MTB molecules and Ba(ii) ions as building blocks results in a three-dimensional framework with 1D channels propagating along the *c* crystallographic axis (see Fig. 5b). The size of the cavities after the subtraction of the van der Waals radii of the selected atoms is 10.7 × 8.7 Å^2^. The topological analysis of the UPJS-9 polymeric framework using the ToposPro program^27^ showed that imitates the fluorite (flu, CaF_2_) structure similar to UPJS-7. The [Ba_3_(μ_2_-COO)_6_(H_2_O)_6_] cluster decorates the calcium(ii) positions and the central methane carbon atom of the MTB ligand can be considered the fluoro vertex in fluorite structure.

Using PLATON analysis the total free pore volume of 2973.5 Å^3^ was found in the structure of compound UPJS-9, which represents 37.1% free space based on the cell volume (8011.1 Å^3^).

### 3.3 Thermal behaviour and frameworks stability study

The thermal behaviour and stability of prepared compounds were investigated using thermogravimetry (TG) and obtained TG curves are shown in Fig. S2a in ESI.‡ The thermogravimetric curve of UPJS-6 displayed no significant mass change in the temperature range 30–210 °C. The mass change corresponding to desolvation process was 16.23% and is in good agreement with calculated value of 16.01% (2 DMF and 4H_2_O molecules). Similar high thermal stability and release of solvent molecules was observed not only for MOF compounds^13^ but also for common chemicals (CuSO_4_·5H_2_O = 50–230 °C; Ca(COO)_2_·H_2_O = 180–200 °C). This phenomenon can be explained by the strong interaction between water molecules and the central atoms. We can only speculate that there is such a strong interaction in UPJS-6, but the main reason for the higher thermal stability (210 °C) is the presence of isolated cages/pores in the framework. The water molecules located in them are trapped and are released only after the collapse of the framework. Mentioned explanation is also in good agreement with the results of the HE-PXRD analysis (see below), where the compound is stable at 200 °C and the polymeric skeleton collapses at 250 °C, allowing water molecules to be released, which was reflected by weight loss on the TG curve. TG curve of UPJS-7 showed a solvent weight loss of 32.49% in the 30–400 °C temperature range (expected 32.29% for 4 DMF and 8H_2_O molecules) and TG curve of UPJS-8 gave a similar solvent weight loss of 29.85% in the 30–300 °C temperature range (expected 29.47% for 4 DMF and 7H_2_O molecules). For compound UPJS-9 TG analysis gave a distinct weight loss of 22.17% in 30–300 °C temperature range corresponding to the loss of 10 water and 2 DMF molecules (expected 22.09%). After the desolvation process, frameworks exhibit high thermal stability up to 500 °C for UPJS-7 and UPJS-9 and 530 °C for UPJS-8. The high thermal stability of polymeric skeletons can be explained by the strong interactions formed between carboxylate oxygen atoms of MTB linker and AE central ions. The TG curves of ethanol exchanged samples UPJS-7′, UPJS-8′ and UPJS-9′ are shown in Fig. S2b in ESI.‡ As can be seen from obtained TG curves, the solvent exchange process has a significant impact on the activation temperature of the compounds. The activation temperatures decrease from 300–400 °C for as-synthesized samples to 150 °C for ethanol exchanged samples. Solvent exchanged samples were further used for gas adsorption measurements.

The framework stability of prepared compounds was studied by high energy powder X-ray diffraction (HE-PXRD) experiments with *in situ* heating and 50 °C steps. Fig. S3a–S6a in ESI‡ show a comparison of experimental HE-PXRD patterns and the patterns calculated from single-crystal X-ray data, which are in good agreement. The framework of UPJS-6 is stable up to 200 °C (see Fig. S3 in ESI‡). Above this temperature, when the solvent molecules are released (see TG), the structure collapses. At this high temperature, the trapped solvent molecules are transformed into the gas phase, because these molecules cannot leave the isolated cages only if the framework collapses, which causes the destruction of the whole crystal structure. Based on the Kitagawa's MOF classification,^31^UPJS-6 can be included in the 1^st^ MOF generation, which is sustained only with guest molecules and shows irreversible framework collapse on the removal of guest molecules. The polymeric skeleton of UPJS-7 (see Fig. S4 in ESI‡) is thermally stable after the release of solvent molecules from the sample and desolvated framework is stable after heating up to 450 °C. Above this temperature, the intensities of the narrow and intense diffraction peaks decrease and at 600 °C formation of amorphous phase was observed. HE-PXRD patterns of UPJS-8 (see Fig. S5b in ESI‡) show similar behaviour to UPJS-7, without changes in the diffraction peak positions during heating. The thermal stability of the polymeric framework of UPJS-8 is higher, up to 500 °C. As the release of the guest molecules located in the cavities (H_2_O and DMF) in UPJS-7 and UPJS-8 occur in the temperature range 30–300 °C (based on TG) and frameworks stability is up to 450 °C (UPJS-7) and 500 °C (UPJS-8) without phase change (based on HE-PXRD), described compounds could be included into the 2^nd^ MOF generation with stable and permanent polymeric frameworks classified by Kitagawa. Compound UPJS-9 exhibits similar thermal stability compared to previous samples. Moreover, a phase transition was observed (see Fig. S6b in ESI‡). The phase representing the as-synthesized compound is stable up to 250 °C. As this temperature is comparable to the end of desolvation step on TG, their release results in the transformation of the compound into a new phase. Novel formed phase is subsequently thermally stable in a temperature range of 300–500 °C. With increasing temperature, the diffraction peaks of the second phase decrease and the appearance of broad peaks, similar to HE-PXRD patterns of previous compounds. Based on the observed results the framework of compound UPJS-9, could be included in the 3^rd^ MOF generation with a flexible framework.

### 3.4 Gas adsorption

To characterize the porosity of prepared MOFs, gas adsorption measurements were performed for nitrogen, argon, hydrogen, methane and carbon dioxide. Before adsorption experiments, the samples were immersed in ethanol for one week to exchange the original solvents in the cavities (DMF and water), for easier activation of the compounds. The samples for gas adsorption experiments were prepared by evacuation at 100 °C for 24 h to remove all guest solvent molecules and activated samples were denoted as UPJS-7′′, UPJS-8′′ and UPJS-9′′.

**Fig. 6:**
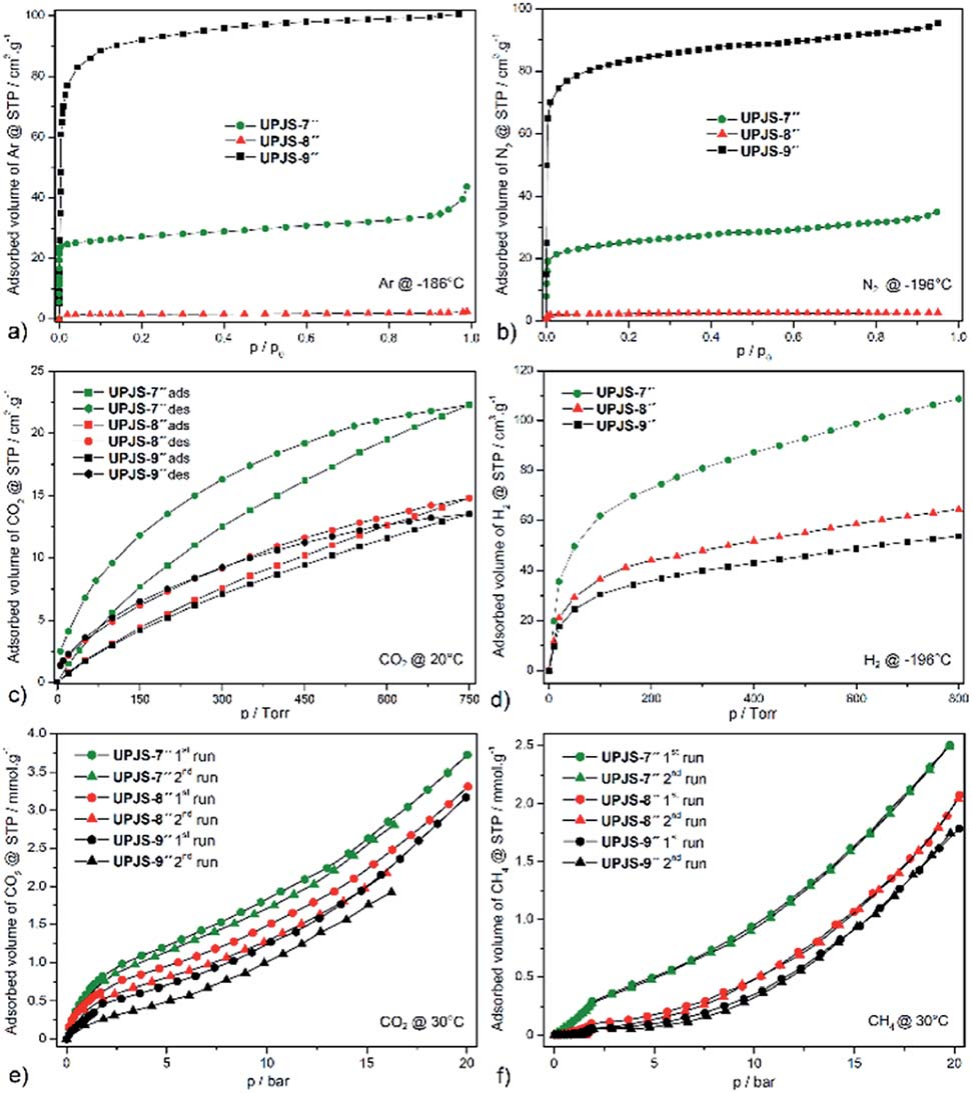
Low pressure adsorption isotherms of (a) argon measured at −186 °C, (b) nitrogen at −196 °C, (c) carbon dioxide at 20 °C and (d) hydrogen at −196 °C. High pressure adsorption isotherm of (e) CO_2_ and (f) CH_4_ at measured at 30 °C and up to 20 bar over prepared compounds.

Textural properties of materials were initially studied by argon (see Fig. 6a) and nitrogen (see Fig. 6b) adsorption measurements at −196 °C and −186 °C, respectively. Based on the nitrogen and argon adsorption isotherms, corresponding surface areas using the BET equation and pore volumes using the DFT were calculated. Compounds UPJS-7′′ and UPJS-9′′ exhibit typical type I nitrogen and argon adsorption isotherms, consistent with their microporous structures. Compounds exhibit BET surface areas of 126 m^2^ g^−1^; pore volume 0.048 cm^3^ g^−1^ (based on Ar) and 103 m^2^ g^−1^; pore volume 0.045 cm^3^ g^−1^ (based on N_2_) for UPJS-7′′, 358 m^2^ g^−1^; pore volume 0.256 cm^3^ g^−1^ (based on Ar) and 320 m^2^ g^−1^; pore volume 0.243 cm^3^ g^−1^ (based on N_2_) for UPJS-9′′. Compound UPJS-8′′ adsorbs only a limited amount of N_2_ and Ar and displayed no porosity. The surface areas of the compounds containing MTB linker are summarized in Table 1. By comparing the values of surface areas, it can be concluded that UPJS-7′′ has the lowest surface area and the surface area of UPJS-9′′ is comparable to the nickel(ii)^33^ and cobalt(ii)^34^ analogues.

**Table 1:** Sorption properties of MOFs containing MTB linker^a^

Compound	Gas	Surface [m^2^ g^−1^]	Pore volume [cm^3^ g^−1^]	Adsorbed CO_2_ @ 1 atm [wt%/°C]	Ref.
{[Sr_3_(μ_7_-MTB)_3/2_]·4DMF·7H_2_O}_*n*_ (UPJS-8)	N_2_	—	—	2.64/20	This work
{[Ca_4_(μ_4_-O)(μ_8_-MTB)_3/2_(H_2_O)_4_]·4DMF·4H_2_O}_*n*_ (UPJS-7)	N_2_, Ar	103, 126	0.045, 0.048	3.92/20	This work
{[Ni_2_(μ_4_-MTB)(κ^4^-L^1^)_2_]·4DMF·8H_2_O}_*n*_	CO_2_	141	0.055	—	32
{[Zn_2_(μ_8_-MTB)(H_2_O)_2_]·3DMF·3H_2_O}_*n*_ (UPJS-2)	N_2_	248	0.089	5.67/0	17
{[Ni_2_(μ_4_-MTB)(κ^4^-L^2^)_2_]·8H_2_O}_*n*_	N_2_	288	0.230	—	33
{[Ba_3_(μ_7_-MTB)_3/2_(H_2_O)_6_]·2DMF·4H_2_O}_*n*_ (UPJS-9)	N_2_, Ar	320, 358	0.243, 0.256	2.41/20	This work
{[Ni_2_(μ_4_-MTB)(κ^4^-L^3^)_2_]·16H_2_O}_*n*_	N_2_	335	0.210		33
{[Co_4_(μ_6_-MTB)_2_(μ_2_-H_2_O)_4_(H_2_O)_4_]·13DMF·11H_2_O}_*n*_	CO_2_	356	0.165	7.02/0	34
{[Cu_2_(μ_8_-MTB)(H_2_O)_2_]·6DEF·2H_2_O}_*n*_	N_2_	526	—	—	35
{[Zn_2_(μ_4_-MTB)(κ^4^-L^1^)_2_]·2DMF·7H_2_O}_*n*_ (UPJS-4)	Ar	644	0.246	10.50/0	18
{[Ni_4_(μ_6_-MTB)_2_(μ_2_-H_2_O)_4_(H_2_O)_4_]·10DMF·11H_2_O}_*n*_ (UPJS-3)	N_2_	700	0.291	12.36/0	19
{[Pb_4_(μ_8_-MTB)_2_(H_2_O)_4_]·5DMF·H_2_O}_*n*_ (UPJS-5)	N_2_	980	0.666	9.30/0	20 and 21
{[Zr_6_(μ_3_-O)(μ_3_-OH)(μ_8_-MTB)_2_(μ_2_-L^4^)_4_(H_2_O)_4_]·*x*DMF·*y*MeOH}_*n*_	N_2_	1390	0.530	—	36

a L^1^ = 1,4,8,11-tetraazacyclotetradecane; L^2^ = 1,3,6,9,11,14-hexaazatricyclodecane; L^3^ = hexaazatricycloeicosane; L^4^ = formate.

The carbon dioxide adsorption isotherms of prepared materials measured at 20 °C show type I behaviour with a hysteresis loop (see Fig. 6c). We suppose that this behaviour arises from the kinetic effect, which may be a contributing factor to the hysteresis because the adsorption/desorption kinetics were very slow, due to a strong interaction between the CO_2_ and framework.^37^ Compounds adsorb CO_2_ with maximal storage capacity of 3.92 wt% (0.927 mmol g^−1^, 22.3 cm^3^ g^−1^) for UPJS-7′′, 2.64 wt% (0.615 mmol g^−1^, 14.8 cm^3^ g^−1^) for UPJS-8′′ and 2.41 wt% (0.561 mmol g^−1^, 13.5 cm^3^ g^−1^) for UPJS-9′′ at 20 °C and 1 atm (760 Torr). The storage capacity of prepared compounds decreases in order UPJS-7′′ > UPJS-8′′ > UPJS-9′′. Calcium MOF (UPJS-7′′) adsorbs the highest amount of CO_2_ from prepared compounds, compared to UPJS-9′′ with the largest surface area (358 m^2^ g^−1^). As was mentioned in the introduction, AE-MOFs made of early members of the metal group are thought to be good candidates based on their gravimetric advantages over commonplace first-row transition metal-based MOFs. Surprisingly, the strontium complex UPJS-8′′ which does not adsorb nitrogen or argon, it adsorbs carbon dioxide. Thus, this compound exhibits a high selectivity for CO_2_ over N_2_ or Ar and can find its further application as a gas separator. High CO_2_/N_2_ selectivity was also observed for Zn-MOF^38^ and UPJS-1.^39^ A likely explanation is that nitrogen molecules cannot enter the pores at −196 °C due to large diffusional resistances, but at 20 °C the additional thermal energy allows the CO_2_ molecules to overcome these resistances. Moreover, higher quadrupole moment of CO_2_ might induce stronger interaction with the framework of UPJS-8′′.

A comparison of the CO_2_ adsorption capacity with reported compounds containing MTB ligand is summarized in Table 1. The adsorbed values of CO_2_ for presented AE-MOF appear to be low, but is not suitable to correlate them with other MTB-containing materials because their CO_2_ adsorption isotherms were measured at 0 °C. A comparison of carbon dioxide capacities can be performed with other MOF compounds that contain alkaline earth metals and measurements were performed under the same conditions, 1 atm and 20 °C. The observed value for UPJS-7′′ is comparable to Ca(SBD) (4.30 wt%; *S*_BET_ = 145 m^2^ g^−1^),^40^ Ca_2_(TIA)_2_(H_2_O)_2_ (4.90 wt%)^41^ but lower than that of compounds Ca_5_(BTB)_2_(HBTB)_2_(H_2_O)_6_ (8.70 wt%; *S*_LANG_ = 914 m^2^ g^−1^),^42^ Mg-MOF-74 (30.1 wt%; *S*_BET_ = 1416 m^2^ g^−1^)^7^ and Mg-DOBDC (25 wt%; *S*_BET_ = 1415 m^2^ g^−1^).^7^ Samples UPJS-8′′ and UPJS-9′′ show lower CO_2_ adsorption capacity values compared to the already published compounds containing strontium or barium ions such as Sr(SBA) (5.10 wt%; *S*_BET_ = 79 m^2^ g^−1^)^43^ and Ba(HBTB) (9.36 wt%; *S*_LANG_ = 879 m^2^ g^−1^).^44^

The hydrogen adsorption isotherms of prepared materials measured at −196 °C are shown in Fig. 6d. Compounds adsorb hydrogen with maximal storage capacity of 3.65 wt% (18.11 mmol g^−1^, 108.7 cm^3^ g^−1^) for UPJS-7′′, 2.17 wt% (10.75 mmol g^−1^, 64.5 cm^3^ g^−1^) for UPJS-8′′ and 1.80 wt% (8.95 mmol g^−1^, 53.7 cm^3^ g^−1^) for UPJS-9′′ at −196 °C and 800 Torr. Observed values of hydrogen storage capacities are relatively high and can be explained by the well-known high affinity of hydrogen to AE metal ions.^13^ Another explanation for the increased affinity of hydrogen for the presented MOFs is the presence of coordinatively unsaturated sites (CUSs) in UPJS-7 and UPJS-9. It is known that MOFs with CUSs offer interesting possibilities for tuning the affinity of these materials towards certain adsorbates, potentially increasing their selectivity and storage capacity. In MOFs with CUSs, at least one available coordination site of the metal centre is occupied by an atom belonging to a solvent molecule after the synthesis. Through activation process using evacuation, or heating, the solvent molecules can be removed, leaving the metal atom with open coordination site. UPJS-7 contains four terminally coordinated water molecules per one cyclotetranuclear cluster [Ca_4_(μ_4_-O)(μ_2_-COO)_8_(H_2_O)_4_], and UPJS-9 contains six water molecules in [Ba_3_(μ_2_-COO)_6_(H_2_O)_6_] cluster (see Section 3.2 Crystal structures). Upon activation of the compounds, these solvent molecules are removed, leading to the formation of CUSs. Formed active sites serve as primary adsorption sites for hydrogen where an interaction central atom-hydrogen is present, thereby increasing the adsorption capacity of hydrogen in MOFs. Although UPJS-9 contains a higher number of CUSs compared to UPJS-7, its hydrogen sorption capacity is lower (53.7 cm^3^ g^−1^ for UPJS-9; 108.7 cm^3^ g^−1^ for UPJS-7). This observation can be explained by the higher relative atomic mass of barium compared to calcium, which is reflected in the resulting values of the volume of adsorbed hydrogen, since in the case of adsorption measurements the amount of gas is given in units of cm^3^ per one gram of support. In compound UPJS-8, no terminally coordinated solvent molecules are present. However, the DFT modeling^23^ has shown that there is one unoccupied orbital on each central atom, which can serve as CUSs (see Fig. S7 in ESI‡). Similar results were observed for other coordination compounds containing strontium ions.^45^

According to the literature data, the H_2_ adsorption capacities were published only for two MTB-containing compounds, namely {[Zn_2_(μ_4_-MTB)(κ^4^-L^1^)_2_]·2DMF·7H_2_O}_*n*_ (UPJS-4)^18^ and {[Ni_2_(μ_4_-MTB)(κ^4^-L^1^)_2_]·4DMF·8H_2_O}_*n*_^32^ (L^1^ = 1,4,8,11-tetraazacyclotetradecane, cyclam). Their hydrogen adsorption capacities at −196 °C were 1.28 and 0.70 wt%, respectively. It indicates that all novel prepared compounds exhibit significantly larger hydrogen uptake.

Compounds UPJS-7′′, UPJS-8′′ and UPJS-9′′ were also tested as gas adsorbents for carbon dioxide and methane at high pressures. Obtained CO_2_ and CH_4_ isotherms measured at 30 °C, pressure up to 20 bar and after two repeated adsorption cycles are depicted in Fig. 6e and f, respectively. The absence of plateaus on the isotherms indicated that the maximal storage capacity for both gases was not achieved and compounds are not saturated by the gases at 20 bar. Both selected probe molecules are nonpolar with different quadrupole moments (CH_4_ (0 C m^2^), CO_2_ (14.3 × 10^−40^ C m^2^)), kinetic diameters (CH_4_ (3.8 Å), CO_2_ (3.3 Å)) and different boiling points ((CH_4_ (−161 °C), CO_2_ (−78 °C))). Described differences may influence the maximal adsorption uptake of gases.

Compounds storage capacities of carbon dioxide at 1 bar and 30 °C were 0.68 mmol g^−1^ (2.99 wt%) for UPJS-7′′, 0.45 mmol g^−1^ (1.98 wt%) for UPJS-8′′ and 0.39 mmol g^−1^ (1.72 wt%) for UPJS-9′′ at 30 °C and 1 bar. A similar trend of adsorbed amounts was also observed for CO_2_ adsorption isotherms measured at 20 °C and 1 atm, described above. With increasing pressure, an increase in the amount of adsorbed CO_2_ was observed. The maximal adsorption uptake and corresponding adsorption capacities were 3.72 mmol g^−1^ (16.4 wt%) for UPJS-7′′, 3.31 mmol g^−1^ (14.6 wt%) for UPJS-8′′ and 3.07 mmol g^−1^ (13.5 wt%) for UPJS-9′′ at 30 °C and 20 bar. For repeated measurements, the branches of adsorption isotherms did not overlap and in the second repetition cycle, the samples adsorbed lower amounts of carbon dioxide compared to the first run. As was described in Section 2.5 Gas adsorption measurements above, the samples at repeated measurements were activated only at 30 °C for 8 hours. Observed results from CO_2_ high pressure adsorption suggest that CO_2_ molecules interact strongly with the frameworks of prepared compounds. A similar observation was demonstrated by the hysteresis loop on CO_2_ adsorption isotherms performed at low pressures.

Methane was also used as a probe molecule for high pressure adsorption and measured isotherms are shown in Fig. 6f. Compounds adsorb CH_4_ with maximal storage capacity of 3.99 wt% (2.49 mmol g^−1^) for UPJS-7′′, 3.32 wt% (2.07 mmol g^−1^) for UPJS-8′′ and 2.86 wt% (1.78 mmol g^−1^) for UPJS-9′′ at 30 °C and 20 bar. The storage capacity of prepared compounds has a similar trend to carbon dioxide and decrease in order UPJS-7′′ > UPJS-8′′ > UPJS-9′′. It could be noted, that the amounts adsorbed for methane are approximately two times lower compared to carbon dioxide. In repeated CH_4_ adsorption measurements, isotherms do not show a significant change in adsorbed volume. This observation indicates that there is no significant interaction between the methane molecules and the polymeric frameworks. Interestingly, the compound does not adsorb CH_4_ at low pressures. The compounds start to adsorb methane up to 0.3 bar for UPJS-7′′, UPJS-8′′ and compound UPJS-9′′ at pressure up to 0.8 bar. On the adsorption isotherms, a breakthrough occurs at a pressure of about 2 bar, where the adsorbed amount of methane is significantly increased. The measured isotherms indicate a possible transformation of the polymeric skeletons, which was confirmed for compound UPJS-9 by HE-PXRD measurements. Measured adsorption results indicate the absence of specific adsorption sites for CH_4_ at the surface of prepared AE-MOFs.

According to the literature data, the high pressure CO_2_ and CH_4_ adsorption measurements were performed for two compounds containing MTB linker. Nickel(ii) MOF with composition {[Ni_4_(μ_6_-MTB)_2_(μ_2_-H_2_O)_4_(H_2_O)_4_]·10DMF·11H_2_O}_*n*_ (UPJS-3)^20^ adsorbed 3.29 wt% (2.05 mmol g^−1^) of CH_4_ and 18.05 wt% (4.1 mmol g^−1^) of CO_2_ and lead(ii) compound with formula {[Pb_4_(μ_8_-MTB)_2_(H_2_O)_4_]·5DMF·H_2_O}_*n*_ (UPJS-5)^20^ adsorbed 3.05 wt% (1.9 mmol g^−1^) of CH_4_ and 19.81 wt% (4.5 mmol g^−1^) of CO_2_ at 30 °C and 21 bar. From the above results, it can be concluded that all prepared AE-MOFs show the same or slightly increased value of methane adsorption capacity and slightly reduced carbon dioxide adsorption capacity.

In the literature, only few MOF compounds containing alkaline earth metals on which the adsorption properties at high pressures and approximately the same conditions have been studied. As an example for comparison could be Mg_2_(DOBDC) (*S*_BET_ = 1495 m^2^ g^−1^, CO_2_, 68.9 wt%, 5 °C, 36 bar; CH_4_, 15.7 wt%, 25 °C, 35 bar);^46^ Ca_2_(AZBZ-TC)(H_2_O)(DMF) (CO_2_, 5.7 wt%, 25 °C, 20 bar)^47^ and Ba_2_(TMA)(NO_3_)(DMF) (*S*_BET_ = 190 m^2^ g^−1^, CO_2_, 11.2 wt%, 0 °C, 70 bar; CH_4_, 1.8 wt%, 0 °C, 70 bar).^48^

## 4. Conclusions

We have prepared and characterized four novel alkaline earth metal–organic frameworks. Structural analysis showed that compounds form 3D frameworks consisting of MTB linker and EA ions, which form different clusters with different SBU. Two different calcium(ii) compounds UPJS-6 and UPJS-7, were prepared using different reaction temperatures. Compound UPJS-6 represents the low-temperature nonporous phase and compound UPJS-7 as the high temperature form exhibited CaF_2_ framework topology with an open structure. Compounds UPJS-8 and UPJS-9 also exhibit porosity with different pore dimensionality. The framework stability of prepared materials was investigated using thermogravimetric analysis and high energy powder X-ray diffraction. The thermal stability of prepared compounds ranged at high temperature 500–530 °C (exception UPJS-6), as a result of strong ionic interaction between AE ions and carboxylates of MTB linker. Moreover, HE-PXRD measurements also confirmed the stability of the frameworks and a phase transition was observed in compound UPJS-9. After the solvent exchange process of as-synthesized samples, materials were thermally activated under vacuum and tested as adsorbents for different probe molecules. Activated materials displayed small surface areas and moderate carbon dioxide storage capacities at low and high pressures, comparable with other MTB-containing compounds. Moreover, CO_2_ adsorption isotherms showed hysteresis at low pressure, respectively reduced adsorbed amount of carbon dioxide at repeated high pressure measurements, indicating an interaction of CO_2_ molecules with the frameworks. The results of methane adsorption measurements showed that the compounds do not adsorb CH_4_ at low pressures (0.3 bar for UPJS-7′′, UPJS-8′′ and 0.8 for UPJS-9′′) and with increasing pressure the adsorbed volume increase. From observed results, it can be concluded that the compounds can be used in the separation process of methane and CO_2_ at low pressures. Moreover, compounds displayed increased adsorbed volume of hydrogen with a sorption capacity of 3.7–1.8 wt% at −196 °C and 800 Torr, which can be explained by the presence of coordinatively unsaturated sites in the MOF's frameworks.

## Conflicts of interest

There are no conflicts to declare.

## Supplementary Material

RA-010-D0RA05145D-s001

RA-010-D0RA05145D-s002
