# Supplementary material for: A series of four novel alkaline earth metal–organic frameworks constructed of Ca(ii), Sr(ii), Ba(ii) ions and tetrahedral MTB linker: structural diversity, stability study and low/high-pressure gas adsorption properties
Source: RSC Adv. 2020 Sep 1;10(54):32323–34. doi: 10.1039/d0ra05145d (PMC9056647; doi:10.1039/d0ra05145d)
Supplement: RA-010-D0RA05145D-s001 [file RA-010-D0RA05145D-s001.pdf]

Electronic Supplementary Material (ESI) for RSC Advances.
This journal is © The Royal Society of Chemistry 2020

Supplementary information

A Series of Four Novel Alkaline Earth Metal-Organic Constructed of Ca(II), Sr(II), Ba(II) Ions and Tetrahedral MTB Linker: Structural Diversity, Stability Study and Low/High-Pressure Gas Adsorption Properties

Miroslav Almáši,[a,*] Vladimír Zeleňák,[a] Róbert Gyepes,[b] Ľuboš Zauška,[a] and Sandrine Bourrelly[c]

[a] *Department of Inorganic Chemistry, Faculty of Science, P. J. Šafárik University, Moyzesova 11, SK-041 54 Košice, Slovak Republic*

[b] *Department of Inorganic Chemistry, Faculty of Science, Charles University, Hlavova 2030/8 CZ-128 43 Prague, Czech Republic*

[c] *Aix-Marseille University, CNRS, MADIREL, F-133 97 Marseille, Cedex 20, France*

*corresponding author: miroslav.almasi@upjs.sk

Tables:

Table S1 Crystal data and structure refinement summary for prepared AE-MOFs.

**Compound**	**UPJS-6**	**UPJS-7**	**UPJS-8**	**UPJS-9**
**Empirical formula**	$C_{58}H_{32}O_{16}Ca_4$	$C_{43,5}H_{32}O_{17}Ca_4$	$C_{43,5}H_{24}O_{12}Sr_3$	$C_{43,5}H_{36}O_{18}Ba_3$
**Molar mass (g.mol^{-1})**	1145.18	987.03	1247.7	1396.85
**Temperature (K)**	150(2)	120(2)	150(2)	150(2)
**Wavelength (nm)**	0.71073	1.54178	0.71073	0.71073
**Crystal size (mm)**	0.47 x 0.44 x 0.30	0.74 x 0.3 x 0.28	0.41 x 0.16 x 0.10	0.30 x 0.16 x 0.13
**Crystal shape**	prism	prism	bar	prism
**Crystal colour**	colourless	colourless	colourless	colourless
**Crystallographic system**	orthorhombic	tetragonal	monoclinic	monoclinic
**Space group**	$Pca2_1$	$I4/m$	$P2_1/n$	$C2/c$
***a* (Å)**	12.4203(6)	13.7591(6)	15.6159(6)	30.7615(16)
***b* (Å)**	22.3330(8)	13.7591(6)	21.5936(8)	11.9378(6)
***c* (Å)**	28.0820(12)	24.7686(15)	28.5465(10)	25.851(2)
***β* (°)**	90	90	102.488(1)	122.448(1)
**Cell volume V_c (Å^{-3})**	7789.5(6)	4689.0(5)	9398.2(2)	8011.0(9)
Z	4	4	4	4
**Density (g.cm^{-3})**	0.976	0.845	0.882	1.158
**Diffractometer**	Nonius KappaCCD	Bruker D8 VENTURE	Nonius KappaCCD	Nonius KappaCCD
**Theta range *θ* (°)**	2.4 ÷ 25.2	4.5 ÷ 57.4	1.2 ÷ 24.0	1.6 ÷ 27.5
**Reflections measured**	66563	15827	41788	27824
**Independent reflections (I ≥ 2σ(I))**	9687	1830	9654	6620
**Absorption coefficient *μ* (mm^{-1})**	0.33	2.41	1.74	1.51
***F*(000)**	2352	1224	2488	2704
**Limiting indices *h***	–15 ÷ 15	–16 ÷ 15	–17 ÷ 17	–39 ÷ 39
**Limiting indices *k***	–27 ÷ 26	–16 ÷ 16	–24 ÷ 20	–15 ÷ 14
**Limiting indices *l***	–34 ÷ 34	–22 ÷ 29	–32 ÷ 26	–33 ÷ 33
**Parameters**	704	103	704	352
**GooF**	1.000	1.059	1.030	1.048
**R_1 (obs.)**	0.057	0.121	0.065	0.039
**wR_2 (obs.)**	0.162	0.409	0.163	0.090

Table S2 Selected bond lengths (Å) and angles (°) in the crystal structure of compound $\{[Ca_4(\mu_8\text{-MTB})_2]\cdot 2DMF\cdot 4H_2O\}_n$ (**UPJS-6**).

Bond lengths (Å)			
Ca1–O5	2.290 (4)	Ca1–O10	2.381 (4)
Ca1–O8	2.264 (4)	Ca1–O13	2.311 (4)
Ca1–O10	2.381 (4)	Ca3–O9	2.338 (3)
Ca1–O13	2.311 (4)	Ca3–O11	2.339 (3)
Ca1–O15	2.285 (3)	Ca4–O1	2.512 (4)
Ca2–O3^{iii}	2.387 (4)	Ca4–O2	2.450 (5)

Ca2–O7^{i}	2.326 (4)	Ca4–O5	2.515 (4)
Ca2–O11^{ii}	2.568 (4)	Ca4–O6	2.457 (4)
Ca2–O12^{ii}	2.447 (4)	Ca4–O9	2.702 (3)
Ca2–O13	2.529 (4)	Ca4–O10	2.490 (3)
Ca2–O14	2.425 (4)	Ca4–O16	2.339 (3)
Ca3–O1	2.305 (4)	Ca4–Ca1	3.7481 (16)
Ca3–O4	2.305 (4)	Ca1–Ca2	4.1509 (17)
Ca1–O5	2.290 (4)	Ca3–Ca4	3.8746 (16)
Ca1–O8	2.264 (4)		
Bond angles (°)			
O5–Ca1–O8	92.71 (14)	O1–Ca3–O9	76.08 (13)
O5–Ca1–O10	74.28 (13)	O1–Ca3–O11	173.89 (15)
O5–Ca1–O13	167.05 (15)	O4–Ca3–O9	169.42 (14)
O5–Ca1–O15	90.24 (14)	O4–Ca3–O11	79.56 (14)
O8–Ca1–O10	166.06 (13)	O9–Ca3–O11	109.06 (13)
O8–Ca1–O13	81.33 (14)	O1–Ca4–O2	50.70 (15)
O8–Ca1–O15	94.39 (14)	O1–Ca4–O5	156.07 (12)
O10–Ca1–O13	112.42 (13)	O1–Ca4–O6	121.88 (12)
O10–Ca1–O15	80.92 (13)	O1–Ca4–O9	66.45 (11)
O13–Ca1–O15	101.62 (13)	O1–Ca4–O10	113.03 (13)
O3^{iii}–Ca2–O7^{i}	92.65 (12)	O1–Ca4–O16	105.89 (15)
O3^{iii}–Ca2–O11^{ii}	92.92 (13)	O2–Ca4–O5	126.16 (16)
O3^{iii}–Ca2–O12^{ii}	143.36 (14)	O2–Ca4–O6	74.34 (13)
O3^{iii}–Ca2–O13	84.74 (13)	O2–Ca4–O9	114.13 (15)
O3^{iii}–Ca2–O14	138.02 (14)	O2–Ca4–O10	163.71 (15)
O7^{i}–Ca2–O11^{ii}	95.03 (13)	O2–Ca4–O16	99.11 (18)
O7^{i}–Ca2–O12^{ii}	101.30 (17)	O5–Ca4–O6	52.65 (12)
O7^{i}–Ca2–O13	97.31 (13)	O5–Ca4–O9	119.12 (12)
O7^{i}–Ca2–O14	93.87 (16)	O5–Ca4–O10	68.61 (12)
O11^{ii}–Ca2–O12^{ii}	52.53 (12)	O5–Ca4–O16	98.04 (14)
O11^{ii}–Ca2–O13	167.54 (13)	O6–Ca4–O10	121.14 (12)
O11^{ii}–Ca2–O14	127.62 (12)	O6–Ca4–O16	99.24 (15)
O12^{ii}–Ca2–O13	126.00 (12)	O9–Ca4–O6	171.48 (12)
O12^{ii}–Ca2–O14	75.11 (12)	O9–Ca4–O10	50.53 (8)
O13–Ca2–O14	53.30 (12)	O9–Ca4–O16	79.00 (11)
O1–Ca3–O4	95.71 (15)	O10–Ca4–O16	84.06 (12)

Symmetry operations: (i) 1−x, −y, −1/2+z; (ii) −1/2+x, 1−y, −1+z; (iii) –x, −y, −1/2+z.

Table S3 Selected bond lengths (Å) and angles (°) in the crystal structure of compound $\{[Ca_4(\mu_4\text{-}O)(\mu_8\text{-}MTB)_{3/2}(H_2O)_4]\cdot 4DMF\cdot 4H_2O\}_n$ (**UPJS-7**).

Bond lengths (Å)			
Ca–O1	2.685 (3)	Ca–O4	2.274 (9)
Ca–O2	2.320 (4)	Ca–O2^{ii}	2.626 (4)
Ca–O3	2.387 (4)	Ca–Ca^{iv}	3.797 (4)
Bond angles (°)			
O1–Ca–O2^{iii}	68.67 (11)	O2^{ii}–Ca–O3	52.14 (14)
O1–Ca–O2^{ii}	64.61 (10)	O2^{ii}–Ca–O2^{iii}	69.51 (17)
O1–Ca–O3^{i}	114.28 (13)	O2^{ii}–Ca–O3^{i}	104.07 (15)
O1–Ca–O4	147.5 (2)	O2–Ca–O4^{i}	86.9 (2)
O2^{iii}–Ca–O2^{i}	80.4 (2)	O2–Ca–O4^{iii}	137.60 (16)
O2–Ca–O2^{ii}	133.1 (2)	O3–Ca–O3^{i}	97.4 (2)
O2^{ii}–Ca–O2^{i}	86.97 (19)	O3^{i}–Ca–O4	86.0 (2)
O2^{i}–Ca–O3^{i}	168.84 (16)	Ca^{V}–O2–Ca	90.0
O2^{i}–Ca–O3	90.64 (14)	Ca^{IV}–Ca–Ca^{V}	90.0

Symmetry operations: (i) x, y, 2–z; (ii) –x, y, 2–z; (iii) –x, y, z; (iv) –x, y, z; (v) x, –y, z.

Table S4 Selected bond lengths (Å) and angles (°) in the crystal structure of compound $\{[Sr_3(\mu_7\text{-}MTB)_{3/2}]\cdot 4DMF\cdot 7H_2O\}_n$ (**UPJS-8**).

Bond lengths (Å)			
Sr1–O1	2.667 (5)	Sr3–O3	2.671 (5)
Sr1–O2	2.707 (3)	Sr2–O10	2.648 (3)
Sr1–O6	2.627 (4)	Sr2–O11	2.603 (6)
Sr1–O7	2.625 (3)	Sr2–O14	2.673 (3)
Sr1–O13	2.607 (5)	Sr2–O15	2.583 (4)
Sr1–O14	2.469 (3)	Sr3–O1	2.644 (2)
Sr1–O16	2.431 (4)	Sr3–O2	2.802 (3)
Sr2–O1	2.615 (2)	Sr3–O4	2.605 (3)
Sr2–O2	2.573 (3)	Sr3–O5	2.588 (4)
Sr2–O3	2.497 (5)	Sr3–O6	2.485 (2)
Bond angles (°)			
O1–Sr1–O6	72.40 (10)	O10–Sr2–O11	49.23 (10)
O1–Sr1–O7	103.21 (12)	O10–Sr2–O15	85.66 (11)
O1–Sr1–O13	49.06 (10)	O11–Sr2–O15	88.23 (11)
O1–Sr1–O14	74.41 (11)	O14–Sr2–O10	79.38 (10)
O1–Sr1–O16	146.88 (11)	O14–Sr2–O11	117.57 (10)
O2–Sr1– O1	62.64 (9)	O14–Sr2–O15	50.08 (10)

O2–Sr1–O6	72.86 (9)	O2–Sr2–O3	81.33 (10)
O2–Sr1–O7	121.97 (11)	O2–Sr2–O10	142.10 (10)
O2–Sr1–O13	111.69 (10)	O2–Sr2–O11	168.52 (10)
O2–Sr1–O14	70.76 (10)	O2–Sr2–O14	69.83 (10)
O2–Sr1–O16	85.71 (11)	O2–Sr2–O15	90.90 (10)
O6–Sr1–O7	50.07 (10)	O3–Sr2–O10	111.95 (11)
O6–Sr1–O13	87.20 (13)	O3–Sr2–O11	95.66 (11)
O6–Sr1–O14	138.94 (10)	O1–Sr3–O2	61.65 (9)
O6–Sr1–O16	89.91 (12)	O1–Sr3–O3	68.43 (9)
O7–Sr1–O13	78.71 (14)	O1–Sr3–O4	115.76 (11)
O7–Sr1–O14	164.83 (12)	O1–Sr3–O5	110.26 (11)
O7–Sr1–O16	84.69 (14)	O1–Sr3–O6	75.04 (10)
O13–Sr1–O14	88.97 (13)	O2–Sr3–O3	74.22 (9)
O13–Sr1–O16	160.53 (13)	O2–Sr3–O4	110.16 (11)
O14–Sr1–O16	105.60 (13)	O2–Sr3–O5	48.61 (10)
O1–Sr2–O2	65.17 (9)	O2–Sr3–O6	73.36 (9)
O1–Sr2–O3	71.52 (10)	O3–Sr3–O4	50.08 (10)
O1–Sr2–O10	84.96 (10)	O3–Sr3–O5	91.84 (10)
O1–Sr2–O11	124.51 (10)	O3–Sr3–O6	139.45 (10)
O1–Sr2–O14	72.03 (10)	O4–Sr3–O5	90.30 (13)
O1–Sr2–O15	122.10 (10)	O4–Sr3–O6	169.15 (12)
O3–Sr2–O14	140.46 (10)	O5–Sr3–O6	84.59 (11)
O3–Sr2–O15	159.63 (11)	Sr2–Sr3–Sr1	60.155 (12)

Table S5 Selected bond lengths (Å) and angles (°) in the crystal structure of compound $\{[Ba_3(\mu_7\text{-MTB})_{3/2}(H_2O)_6]\cdot 2DMF\cdot 4H_2O\}_n$ (**UPJS-9**).

Bond lengths (Å)			
Ba1–O1	2.740 (3)	Ba2–O11	2.807 (3)
Ba1–O4	2.671 (3)	Ba2–O13	2.849 (5)
Ba1–O5	2.700 (2)	Ba1–Ba2	4.4208 (4)
Ba2–O1	2.850 (3)	C1–O1	1.257 (4)
Ba2–O2	2.833 (3)	C1–O2	1.256 (5)
Ba2–O3	2.693 (4)	C13–O3	1.245 (6)
Ba2–O5	2.848 (3)	C13–O4	1.250 (6)
Ba2–O6	2.871 (2)	C20–O5	1.256 (5)
Ba2–O7	2.908 (3)	C20–O6	1.257 (5)
Ba2–O10	2.812 (2)		
Bond angles (°)			

O1–Ba1–O1^{i}	146.76 (14)	O2–Ba2–O13	85.72 (17)
O1–Ba1–O4	69.56 (11)	O3–Ba2–O5	89.27 (10)
O1–Ba1–O4^{i}	141.90 (10)	O3–Ba2–O6	92.07 (10)
O1–Ba1–O5	72.90 (8)	O3–Ba2–O7	143.28 (12)
O1–Ba1–O5^{i}	111.92 (8)	O3–Ba2–O10	78.62 (11)
O4–Ba1–O4^{i}	80.14 (17)	O3–Ba2–O11	123.82 (11)
O4–Ba1–O5	85.41 (11)	O3–Ba2–O13	146.31 (14)
O4–Ba1–O5^{i}	82.41 (11)	O5–Ba2–O6	45.34 (8)
O5–Ba1–O5^{i}	164.07 (15)	O5–Ba2–O7	92.91 (9)
O1–Ba2–O2	45.84 (8)	O5–Ba2–O10	116.59 (9)
O1–Ba2–O3	76.61 (11)	O5–Ba2–O11	146.65 (11)
O1–Ba2–O5	69.12 (8)	O5–Ba2–O13	74.41 (15)
O1–Ba2–O6	113.78 (7)	O6–Ba2–O7	64.91 (9)
O1–Ba2–O7	137.77 (11)	O6–Ba2–O10	72.88 (9)
O1–Ba2–O10	154.52 (10)	O6–Ba2–O11	127.49 (10)
O1–Ba2–O11	111.26 (11)	O6–Ba2–O13	96.51 (16)
O1–Ba2–O13	70,10 (14)	O7–Ba2–O10	67.70 (11)
O2–Ba2–O3	74.54 (12)	O7–Ba2–O11	63.84 (11)
O2–Ba2–O5	114.85 (8)	O7–Ba2–O13	68.29 (15)
O2–Ba2–O6	157.25 (8)	O10–Ba2–O11	78.09 (12)
O2–Ba2–O7	135.48 (10)	O10–Ba2–O13	135.01 (14)
O2–Ba2–O10	120.70 (9)	O11–Ba2–O13	74.76 (15)
O2–Ba2–O11	75.00 (11)	Ba2–Ba1–Ba2^{i}	165.579 (13)

Symmetry operations: (i) –x, y, 1/2–z.

Figures:

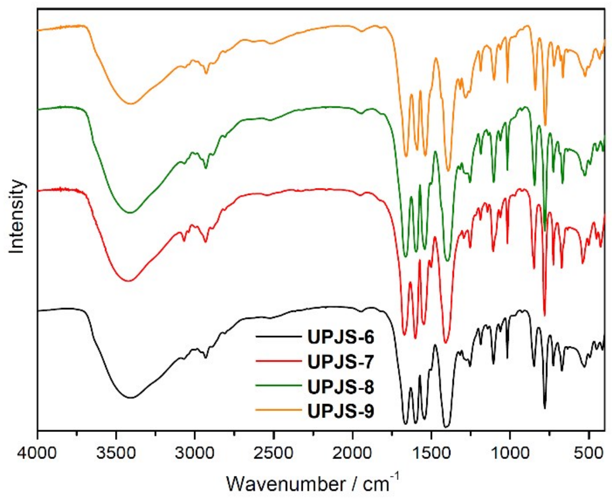

Fig. S1 Infrared spectra of prepared compounds.

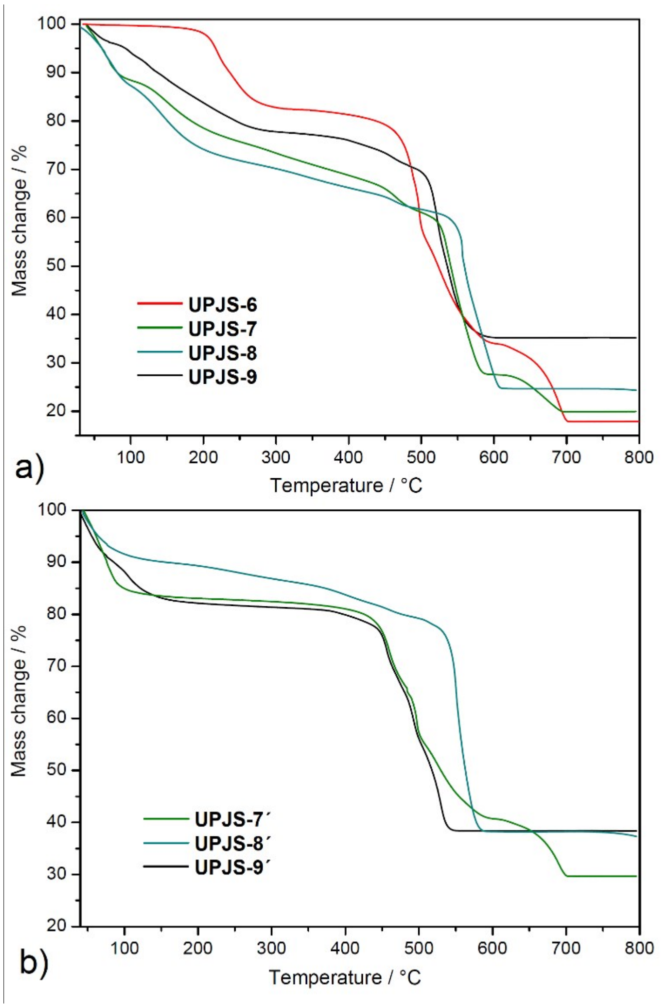

Fig. S2 TG analytical curves of a) as-synthesized samples and b) ethanol-exchanged materials.

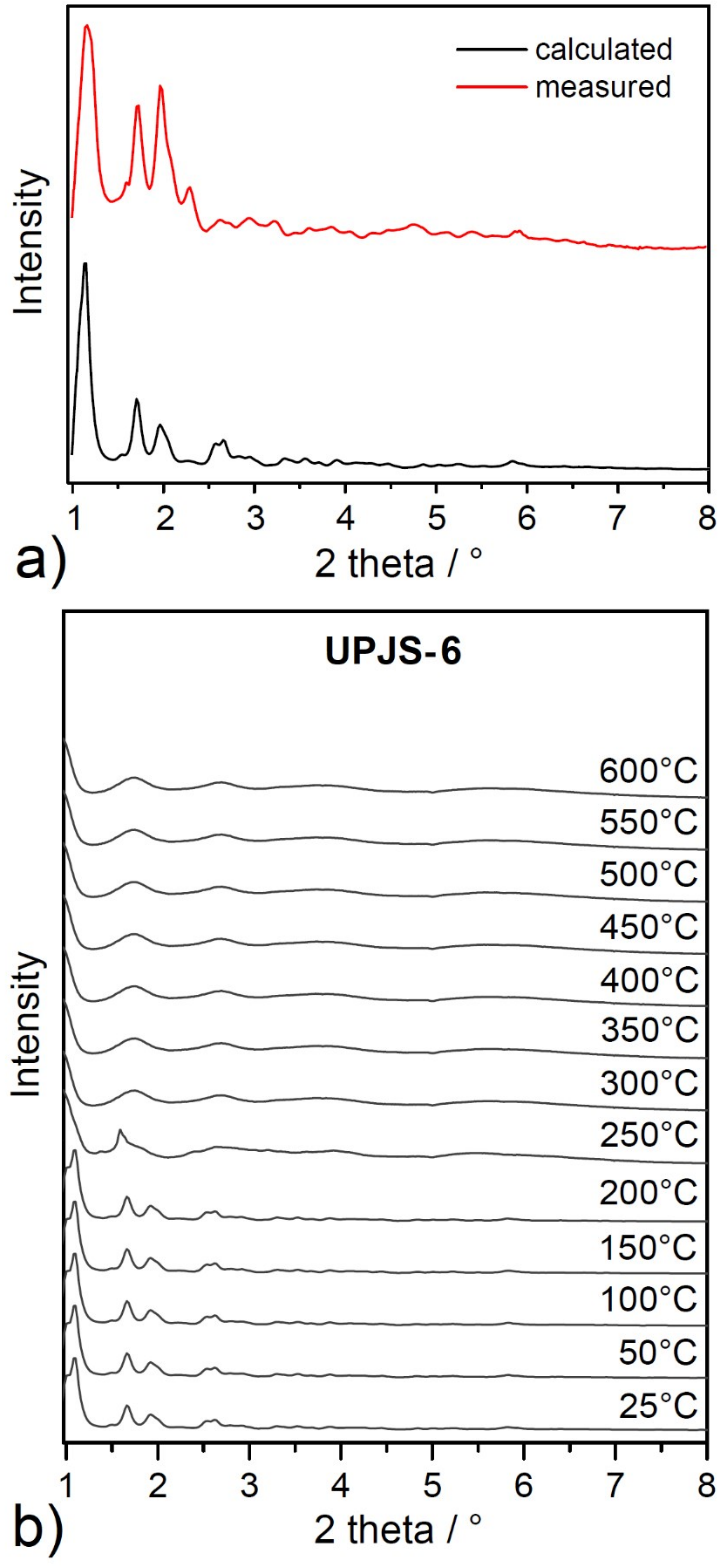

Fig. S3 a) The comparison of measured HE-PXRD pattern of as synthesized sample (red line) and the pattern calculated from single X-ray diffraction data (black line). b) HE-PXRD patterns of compound **UPJS-6** measured during *in-situ* heating in a temperature range of 25-600°C with 50°C step and 2θ range 0.9-8° in an atmosphere of argon.

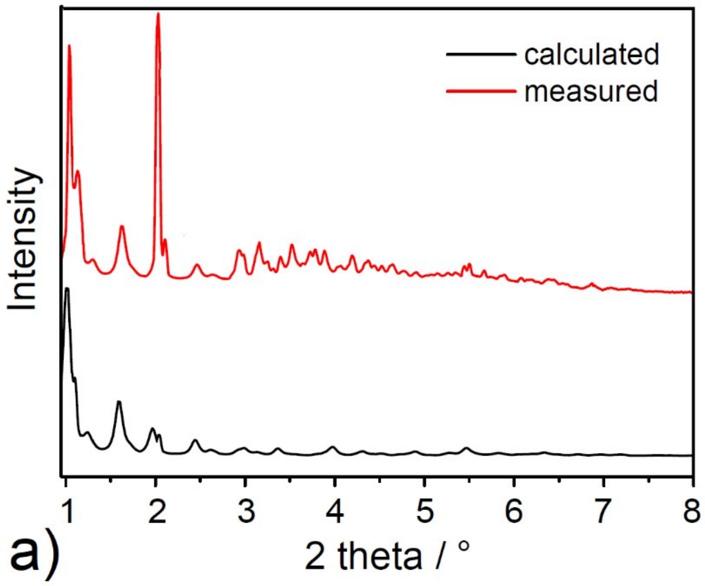

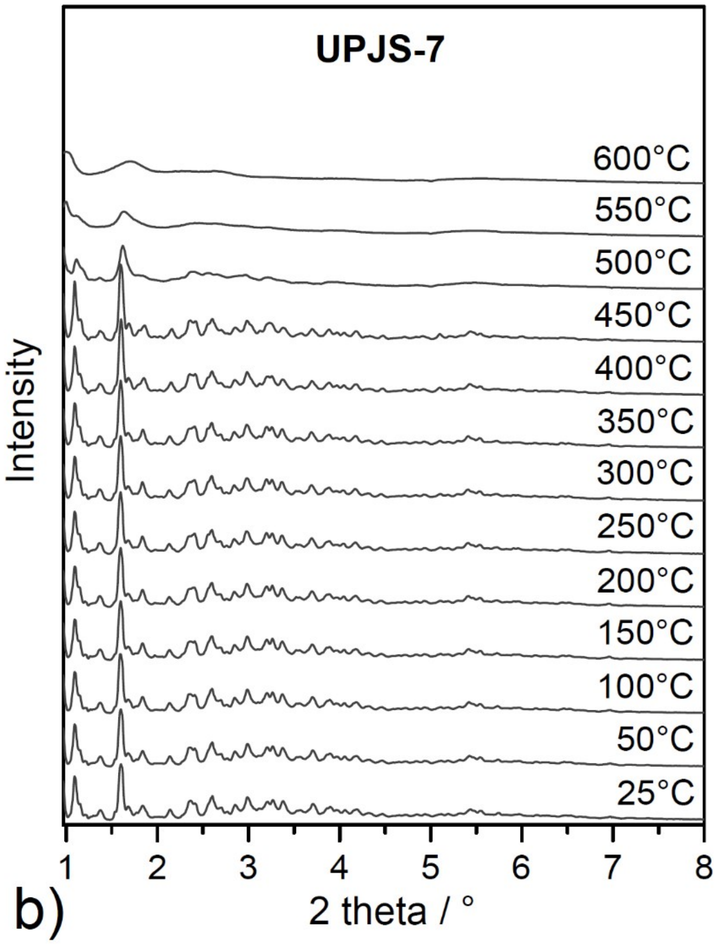

Fig. S4 a) The comparison of measured HE-PXRD pattern of as synthesized sample (red line) and pattern calculated from single X-ray diffraction data (black line). b) HE-PXRD patterns of compound **UPJS-7** measured during *in-situ* heating in a temperature range of 25-600°C with 50°C step and 2θ range 0.9-8° in an atmosphere of argon.

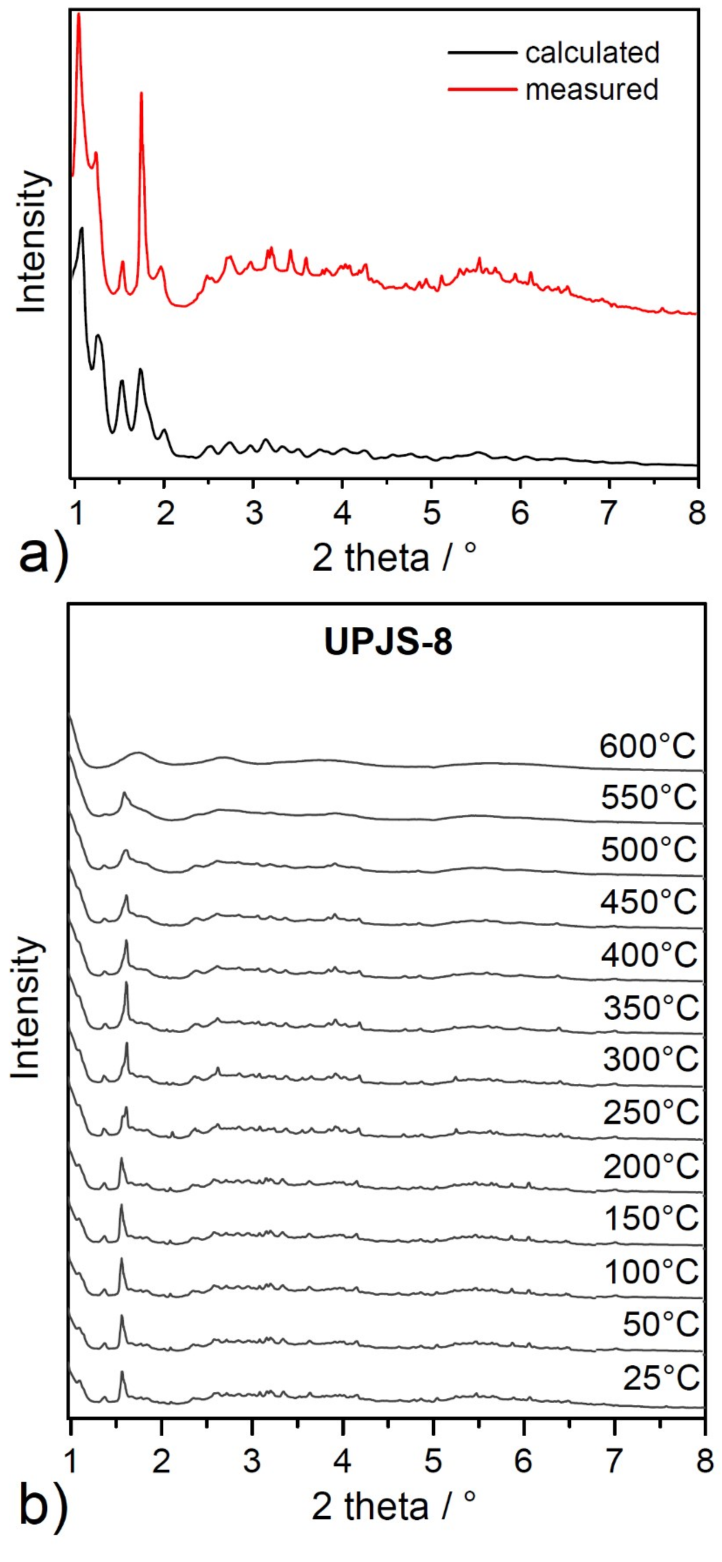

Fig. S5 a) The comparison of measured HE-PXRD pattern of as synthesized sample (red line) and the pattern calculated from single X-ray diffraction data (black line). b) HE-PXRD patterns of compound **UPJS-8** measured during *in-situ* heating in a temperature range of 25-600°C with 50°C step and 2θ range 0.9-8° in an atmosphere of argon.

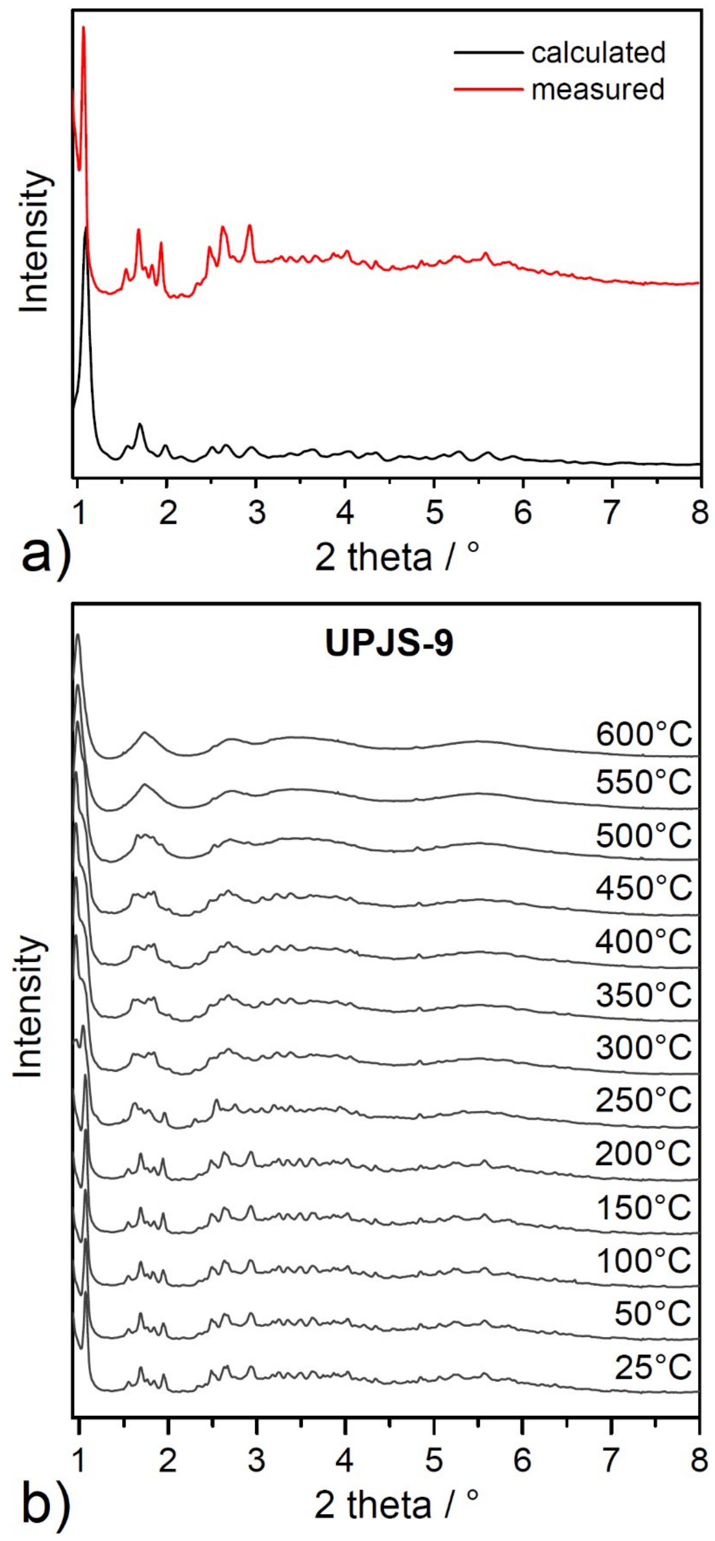

Fig. S6 a) The comparison of measured HE-PXRD pattern of as synthesized sample (red line) and the pattern calculated from single X-ray diffraction data (black line). b) HE-PXRD patterns of compound **UPJS-9** measured during *in-situ* heating in a temperature range of 25-600°C with 50°C step and 2θ range 0.9-8° in an atmosphere of argon.

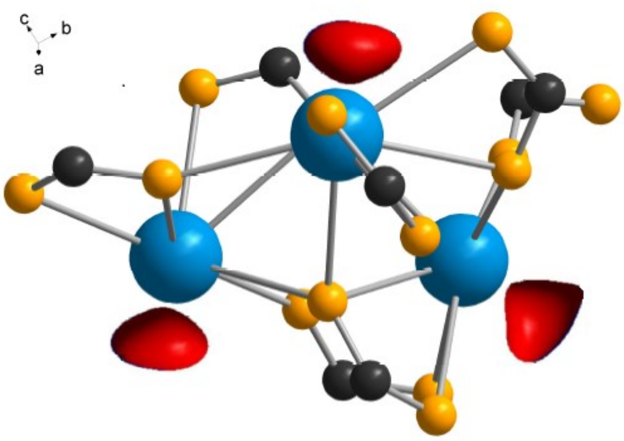

Fig. S7 A view of unoccupied orbitals (red colour) located on strontium ions in compound **UPJS-8**.
